# Photochemical and Thermal Activation of a Diarylbismuth Azide

**DOI:** 10.1002/chem.71556

**Published:** 2026-08-13

**Authors:** Giacomo Pugliese, Oliver Pichl, Gregor Schnakenburg, Peter Vöhringer, Luis I. Domenianni, Alessandro Bismuto

**Affiliations:** ^1^ Institute of Inorganic Chemistry University of Bonn Bonn Germany; ^2^ Clausius‐Institut für Physikalische und Theoretische Chemie University of Bonn Bonn Germany

**Keywords:** bismuth azides, main‐group chemistry, nitrene generation, ultrafast spectroscopy

## Abstract

Azides are widely employed in synthesis as convenient nitrene precursors, particularly in transition‐metal chemistry, where metal–nitrene intermediates enable diverse C─H amination, aziridination, and atom‐transfer processes. In stark contrast, main‐group azides, especially bismuth ones, are underrepresented and have not been fully exploited as nitrene sources. Herein, we report the synthesis and full characterization of a diarylbismuth monoazide supported by a diphenylsulfone‐based dianionic ligand. The complex is robust toward air, moisture, and elevated temperatures, enabling detailed photochemical and chemical studies. Ultrafast UV‐pump/mid‐IR‐probe spectroscopy demonstrates that 266 nm excitation triggers rapid azide‐to‐ligand charge transfer and loss of the azide antisymmetric stretching band. FTIR‐monitored trapping with *t*‐butyl isonitrile produces a new absorption band in the heterocumulene region, suggesting the formation of a nitrene intermediate. Alternatively, Lewis‐acid activation with B(C_6_F_5_)_3_ produces a stable adduct that undergoes thermal N_2_ extrusion, transmetalation, and cycloaddition to form a tetrazaborole dimer. These divergent activation pathways establish bismuth azides as viable nitrene precursors and expand the reactivity landscape of heavy *p*‐block chemistry.

## Graphical abstract

We report the synthesis and characterization of a novel diarylbismuth azide and its divergent reactivity upon thermal or photochemical activation. These findings contribute expanding the reactivity landscape of heavy pnictogen compounds and sets the basis for further investigations.

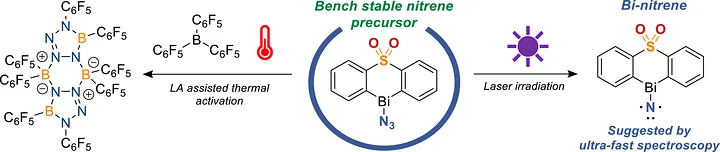

## 1 Introduction

Transition metal (TM) complexes featuring a monocoordinated N atom are responsible for a variety of useful transformations in modern chemistry, including C─H amination, aziridination of alkenes, and nitrogen‐atom transfer (Scheme 1A) [1, 2, 3, 4, 5, 6, 7, 8]. A pioneering work was reported by Meyer and coworkers with the first synthesis of an osmium nitride complex capable of insertion into C─C double bonds to yield the corresponding azaallenium complexes [6, 9]. Subsequently, Protasiewicz and coworkers synthesized a molybdenum nitride complex which was later indicated as key intermediate in the incorporation of N_2_ into organic substrates [7, 8]. More recently, Schneider and coworkers synthesized a platinum nitrene complex with a triplet ground state that undergoes N‐atom insertion into C─H, B─H, and B─C bonds [4, 10]. In this context, ultra‐fast spectroscopy represents a powerful tool that allowed elucidation of the mechanism of formation of such reactive intermediates. In particular, ultra‐fast pump–probe experiments have directly tracked the loss of N_2_ on femtosecond to picosecond timescales [11, 12, 13]. The accumulated experimental and theoretical evidences indicated that metallonitrene formation from azides proceeds through azide‐centered diradical excited states, with N_2_ loss occurring adiabatically after spin reorganization.

**SCHEME 1:**
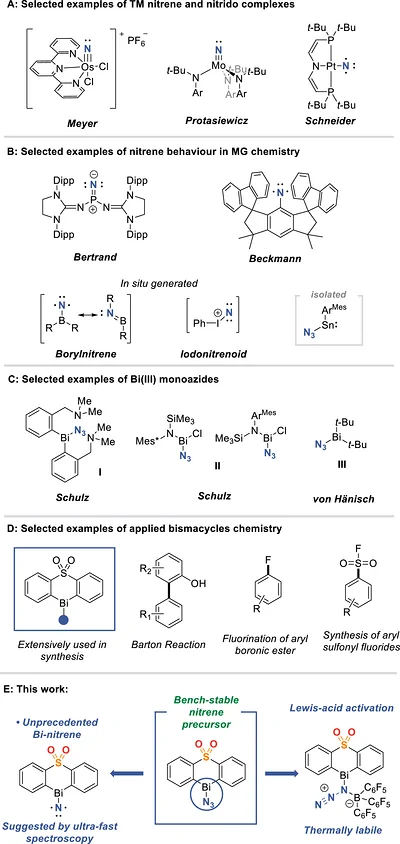
(A) Selected examples of TM‐nitrene and ‐nitrido complexes; (B) Selected examples of nitrene‐like behavior in MG chemistry; (C) Selected examples of reported bismuth monoazides; (D) Selected examples of applications of Suzuki‐type bismacycles; (E) This work: synthesis, characterization, and reactivity of a new diarylbismuth monoazide.

In main‐group (MG) chemistry by contrast, isolated compounds featuring a monocoordinated N remain comparatively rare relative to their TM analogs, with main examples involving lighter MG elements such as phosphorous or carbon (Scheme 1B) [14, 15, 16]. Specifically, Bertrand and coworkers reported the synthesis and isolation of an electronically stabilized phosphinonitrene which served as a nitrogen atom transfer [14]. More recently, Beckmann and coworkers exploited the steric demand of the M^S^Fluind ligand scaffold to isolate a crystalline aryl nitrene [15]. Beyond these examples, MG nitrenes and nitrenoid intermediates have emerged as powerful synthons despite the lack of structural characterization. *In situ* generated iodine and boron based nitrenoids are prominent examples: such species have been exploited in several efficient synthetic protocols for aziridation, skeletal editing, and C─H bond insertion (Scheme 1B) [17, 18, 19, 20, 21, 22, 23]. Recently, other heavy MG azido species have been recognized for their ability to generate analogues reactive intermediates and engage in related bond‐activation pathways [24, 25, 26, 27]. As an example, an organo‐tin azide was reported by Power and coworkers to react with tris‐pentafluorophenyl borane (BCF) under UV light irradiation releasing nitrogen and forming the product of the formal N insertion into the B─C bond [24].

Among MG elements, bismuth occupies a unique position: its diverse and accessible oxidation states, and the strong relativistic stabilization, enable reactivity reminiscent of late transition metals, offering new opportunities for MG mediated group‐transfer processes [28, 29, 30, 31, 32, 33, 34]. Despite this premise, structurally well‐defined bismuth azido complexes remain rare. Most known examples belong to inorganic or ionic Bi(III)—N_3_ systems, of the form [Bi(N_3_)_n_]^(n−3)−^ (*n* = 3, 4, 6) which were structurally characterized by Schulz and coworkers, and display high coordination numbers and pronounced thermal and mechanical instability [35]. This chemistry was later extended to binary pnictogen azides and donor‐stabilized variants such as [Bi(N_3_)_5_(dmso)]^2^^−^ and [Py_2_Bi(N_3_)_3_] (Py = pyridine) instead a limited number of ligand‐supported complexes, including amidinate‐stabilized LBi(N_3_)_2_ and L_2_BiN_3_ derivatives, have also been reported, but their reactivity or deep electronic investigation was not fully disclosed (Scheme 1C) [36, 37, 38, 39, 40, 41, 42, 43]. A. Schulz and co‐workers gave a major contribution in this area by synthesizing the first diarylbismuth monoazide (**I**), which to the best of our knowledge remains the only diarylbismuth azide characterized in the solid‐state. In their quest for the synthesis of nitrogen rich compounds, they also reported amide substituted sterically protected bismuth mono‐ and diazides (**II**). Additionally, von Hänisch and coworkers have more recently put efforts in the structural characterization of an alkylbismuth monoazide with two *t*‐butyl substituents (**III**). Organobismuth azides generally showcase good stability toward high temperatures and atmospheric conditions, yet the monodentate nature of their ligand motifs represents a liability for the development of a selective reactivity based on the Bi─N_3_ fragment. This underscores the need for a robust ligand scaffold that not only stabilizes the bismuth─azide bond under demanding conditions but also facilitates its site‐selective activation into reactive intermediates. This challenge emerges at a particularly exciting moment in the field where some robust ligand scaffolds have been instrumental to unlock stoichiometric and catalytic bismuth chemistry. Pivotal examples are the arylation reactions reported by Ball and co‐workers [28, 32, 44, 45, 46, 47] as well as the Bi(III)/Bi(V) and Bi(I)/Bi(III) redox cycles described by Cornella for oxidative coupling, fluorination, and amination reactions (Scheme 1D) [30, 32, 46, 47, 48, 49].

Here, we report the isolation and comprehensive characterization of a bismuth─azide complex supported by a phenyl‐based di‐anionic ligand framework (Scheme 1E). Although the ligand framework does not include any sterically demanding fragment that could confer kinetic stabilization, the resulting Bi─N_3_ fragment exhibits marked robustness toward high temperatures, air and moisture, and proved surprisingly resilient toward a variety of reaction conditions (vide infra). Upon 266 nm laser irradiation, infrared ultrafast and quenching experiments are consistent with the involvement of a transient bismuth nitrene intermediate. The anionic ligand also enables selective Lewis‐acid activation with BCF, producing a reactive intermediate that releases N_2_ upon heating to afford an iminoborane derivative. This divergent reactivity significantly expands the reactivity landscape of bismuth chemistry and highlights the untapped potential of heavy main‐group elements for controlled nitrene generation and functionalization.

## 2 Results and Discussion

Bismuth compounds bearing diphenylsulfone based ligands proved to be particularly stable and easy to handle since their first report by Suzuki in 1992 [48, 49]. Thus, we identified this scaffold as the best candidate for the present investigation. Accordingly, we used compound **1** as a starting material for the installation of the azide motif. Generally, bismuth iodides undergo smooth halogen–azide exchange when mixed with AgN_3_. However, to limit our usage of sensitive explosives, we developed an iodine‐catalyzed protocol to accomplish such transformations using NEt_4_N_3_ as a metathesis partner (Figure 1, and Table S1 for further details). We isolated compound **2** as a white powder in 79% yield, and obtained single crystals suitable for X‐ray measurements upon crystallization at −18°C of a saturated DCM:*i*‐PrOH solution (2:1 v/v). Compound **2** remains stable in air up to 242°C, consistently with established empirical safety guidelines for organic azides, as the (C+O)/N ratio of 4.7 in **2** significantly exceeds the commonly cited hazard threshold of 3.0.

**FIGURE 1:**
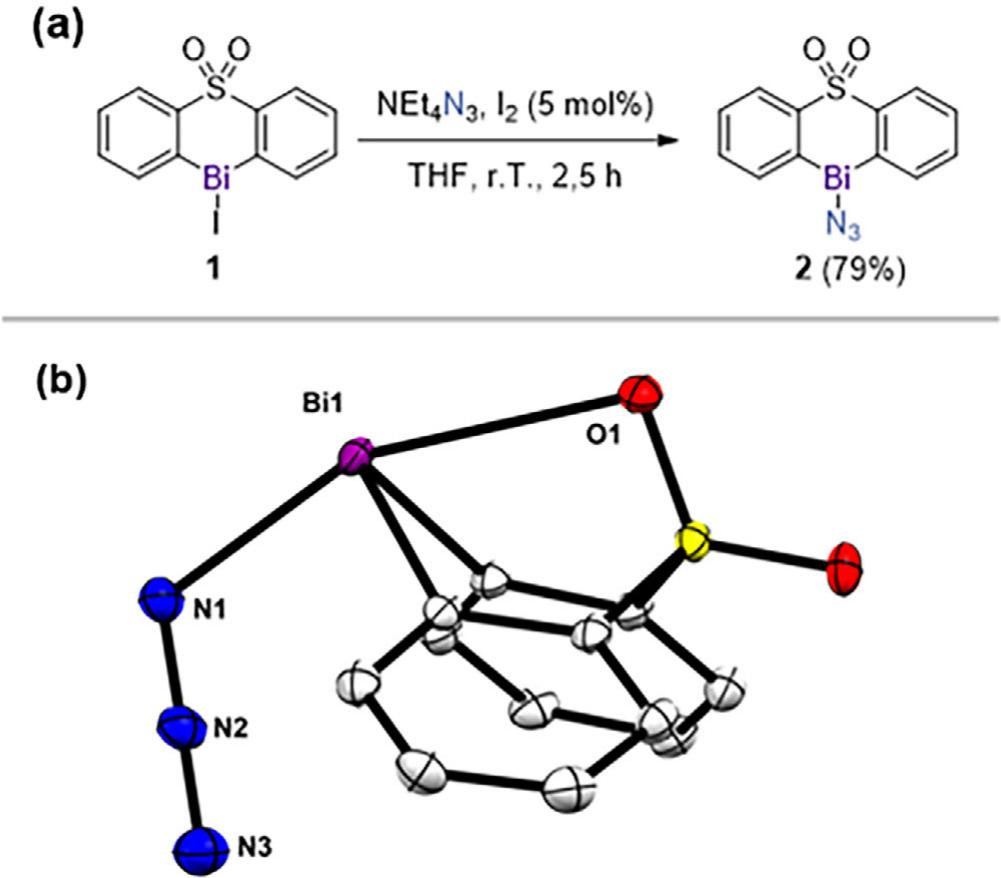
(a) Reaction scheme for the synthesis of **2**; (b) Single‐crystal x‐ray structure of complex **2**. Thermal ellipsoids are set at 50% probability. Hydrogens omitted for clarity. Selected bond lengths (Å) and angles (°): Bi1─N1 = 2.265(4); N1─N2 = 1.203(5); N2─N3 = 1.161(6); Bi1─O1 = 2.751(3); Bi1─N1─N2 = 117.1(3); N1─N2─N3 = 179.1(3).

The Bi1─N1 distance in **2** is 2.265(4) Å which is close to the sum of the covalent radii (2.20 Å), and is shorter than that of the only other analog example of a diarylbismuth monoazide for which a crystal structure is reported (Scheme 1C, **I**, Bi1─N1 = 2.413(2) Å) [36]. Extending the discussion to non‐arylated monomeric bismuth monoazides, the Bi1—N1 bond length is instead longer than average (2.218(2) Å [37], 2.217(8) Å [41]). The Bi‐N1‐N2 angle in **2** is 117.1(3)°, which is slightly narrower than the same angle in **I** (Bi─N1─N2 in **I** = 120.6(2)°), possibly indicating a higher charge density on N1 as compared to **I**. The N1─N2─N3 fragment appears to be close to linearity (N1─N2─N3 = 179.1(3)°), and the difference between the lengths of the N1─N2 bond and N2─N3, which correlates with the triple bond character between N2─N3, is higher in **2** (0.042 Å) than it is for **I** (0.036 Å). Unfortunately, our attempt to further characterize the azide group via ^1^H‐^15^N HMBC NMR experiments was not successful.

With the compound in hand, we next examined its reactivity. Attempts to engage **2** in the Staudinger reaction with triphenylphosphine or tris‐*t*‐butylphosphine were unsuccessful, even under forcing conditions. Similarly, amidation of *p*‐nitrobenzaldehyde failed under both photochemical and thermal conditions. Inspired by a report from Fokin and coworkers, in which bismuth acetylides bearing Suzuki‐type ligand frameworks were used to generate triazole anion equivalents in copper‐catalyzed reactions with organic azides [50], we also explored an analogous transformation with inverted partners. Accordingly, compound **2** was combined with phenylacetylene in the presence of a copper catalyst, but no reaction was observed. We further investigated click‐type reactivity using highly activated multiple‐bond systems, such as maleic anhydride and other electrophiles; however, no selective product formation but only unreacted starting material was observed.

As we could not identify the reason for such stability, we decided to gain more information about the nature of this fragment utilizing ultra‐fast spectroscopy. This technique has been previously employed in transition metal–azides to elucidate the formation of potential nitrene species [12]. However, such technique has never been employed in the case of bismuth azides. Heavy p‐block elements such as bismuth occupy an intermediate position between MG and TM regimes: while lacking the dense manifold of metal‐centered d‐states characteristic of TM, bismuth combines substantial spin–orbit coupling with polarized covalent bonding, offering a unique platform for stabilizing and harnessing nitrene intermediates generated by azide photolysis, while retaining key features of ligand‐centered photochemistry. Understanding how these distinct chemical contexts influence nitrene generation is therefore central to the development of new photochemical nitrene transfer strategies.

The stationary UV–vis electronic absorption spectrum of compound **2** in CHCl_3_ at room temperature is reproduced in Figure 2. The spectrum exhibits a gradual absorption onset at ca. 300 nm, followed by a plateau region around 266 nm and a pronounced increase in absorption at higher energies. Solvent absorption prevents reliable measurement of the spectrum for shorter wavelengths. To elucidate the electronic transitions underlying the UV–vis spectrum, time‐dependent density functional theory (TD‐DFT) calculations were performed. Following geometry optimization of **2** in its singlet electronic ground state, its electronically excited states were calculated at the relaxed S_0_ geometry. The resulting stick‐spectrum (oscillator strength versus excitation wavenumber) was then convoluted with a Gaussian line‐shape function (FWHM = 4000 cm^−1^) to phenomenologically account for spectral broadening effects. After application of a small empirical energy shift, the resulting in silico spectrum reproduces the experimental absorption profile very well (Figure 2a).

**FIGURE 2:**
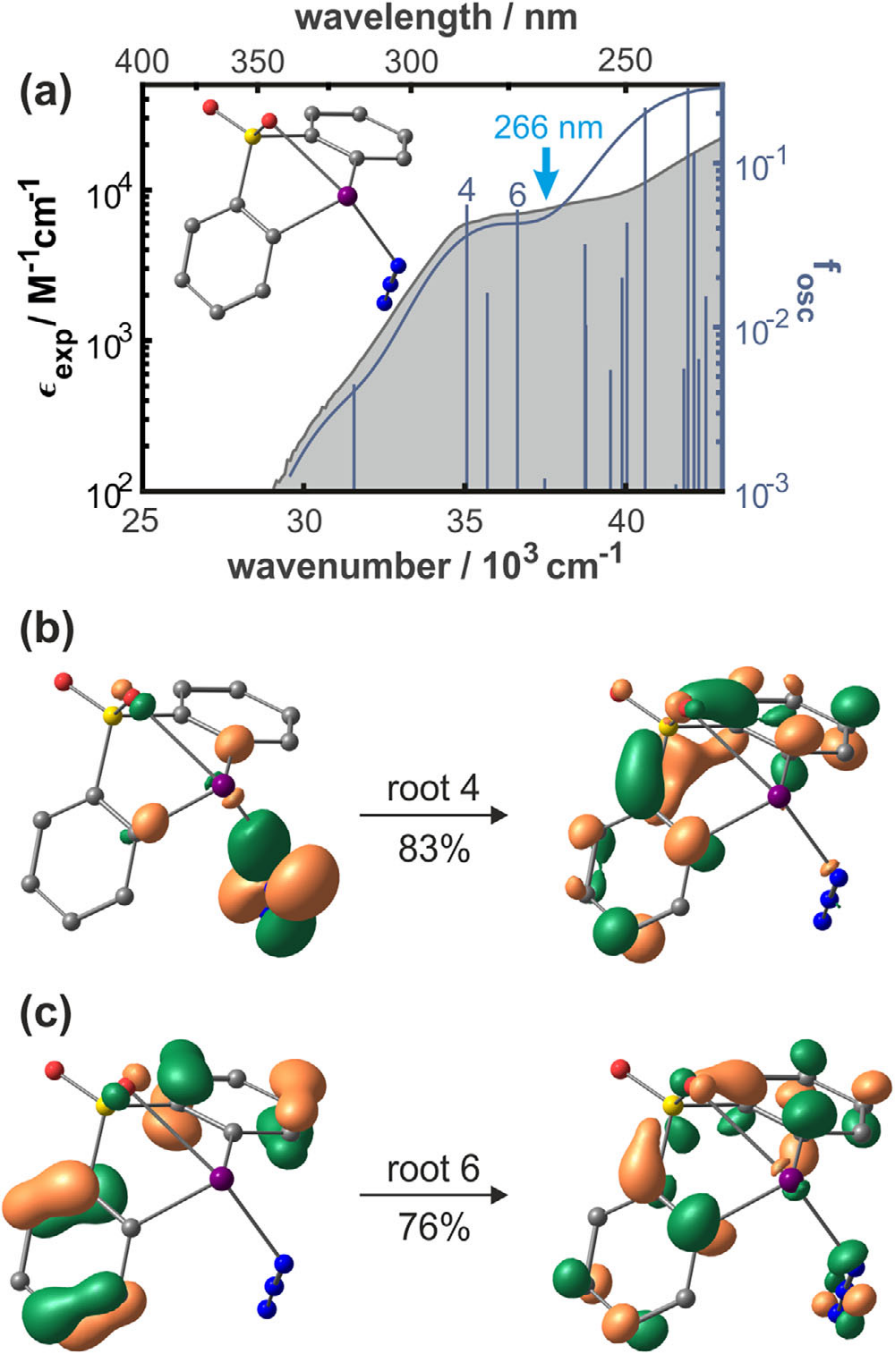
(a) Experimental UV–vis spectrum of **2** in CHCl_3_ at 293 K (shaded gray) and TD‐DFT‐computed spectra (blue) at the PBE0D3/def2TZVP level of theory. The vertical arrow (light blue) specifies the spectral location of the 266 nm‐pump‐pulses. (b, c) Natural transition orbitals (NTOs, holes on the left and particles on the right) associated with the S6 and S4‐states, respectively. Hydrogens omitted for clarity.

This close agreement between experiment and theory provides a reliable electronic‐structure framework for interpreting the subsequent ultrafast spectroscopic experiments and, critically, for assigning the nature of the initially populated excited states. The UV/MIR transient spectroscopy experiments employed an excitation wavelength of 266 nm, corresponding to a region of the absorption spectrum dominated by excitations associated with roots 4 and 6 (Figure 2b,c, respectively). A natural transition orbital (NTO) analysis reveals that these states are primarily an azide‐to‐diphenylsulfone ligand‐to‐ligand charge‐transfer (LLCT) transition (root 4) and a diphenylsulfone‐centered intraligand π–π* excitation (root 6).

The stationary Fourier‐transform infrared (FTIR) spectrum of **2** in a room temperature CHCl_3_ solution is depicted in Figure 3c (gray) and S2 (blue), with emphasis on the azide antisymmetric stretching region between 1900 and 2150 cm^−1^. A single absorption band corresponding to the antisymmetric stretching vibration of the azido ligand is centered at 2058 cm^−1^, with a peak extinction coefficient of 2705 M^−1^ cm^−1^. For comparison, the free azide anion in CHCl_3_ solution (see Figure S2 (orange)) exhibits its antisymmetric stretching absorption at 2012 cm^−1^ with a peak extinction coefficient of 2423 M^−1^ cm^−1^. This behavior is consistent with previous reports, which showed that coordination to a transition‐metal center leads to a substantial frequency shift of the azide antisymmetric stretch while largely preserving the associated IR transition dipole moment [12, 51, 52, 53, 54]. As a consequence, the azide antisymmetric stretch serves as a sensitive reporter of the ligand's electronic state, making it ideally suited for tracking photoinduced charge redistribution and bond activation in real time.

**FIGURE 3:**
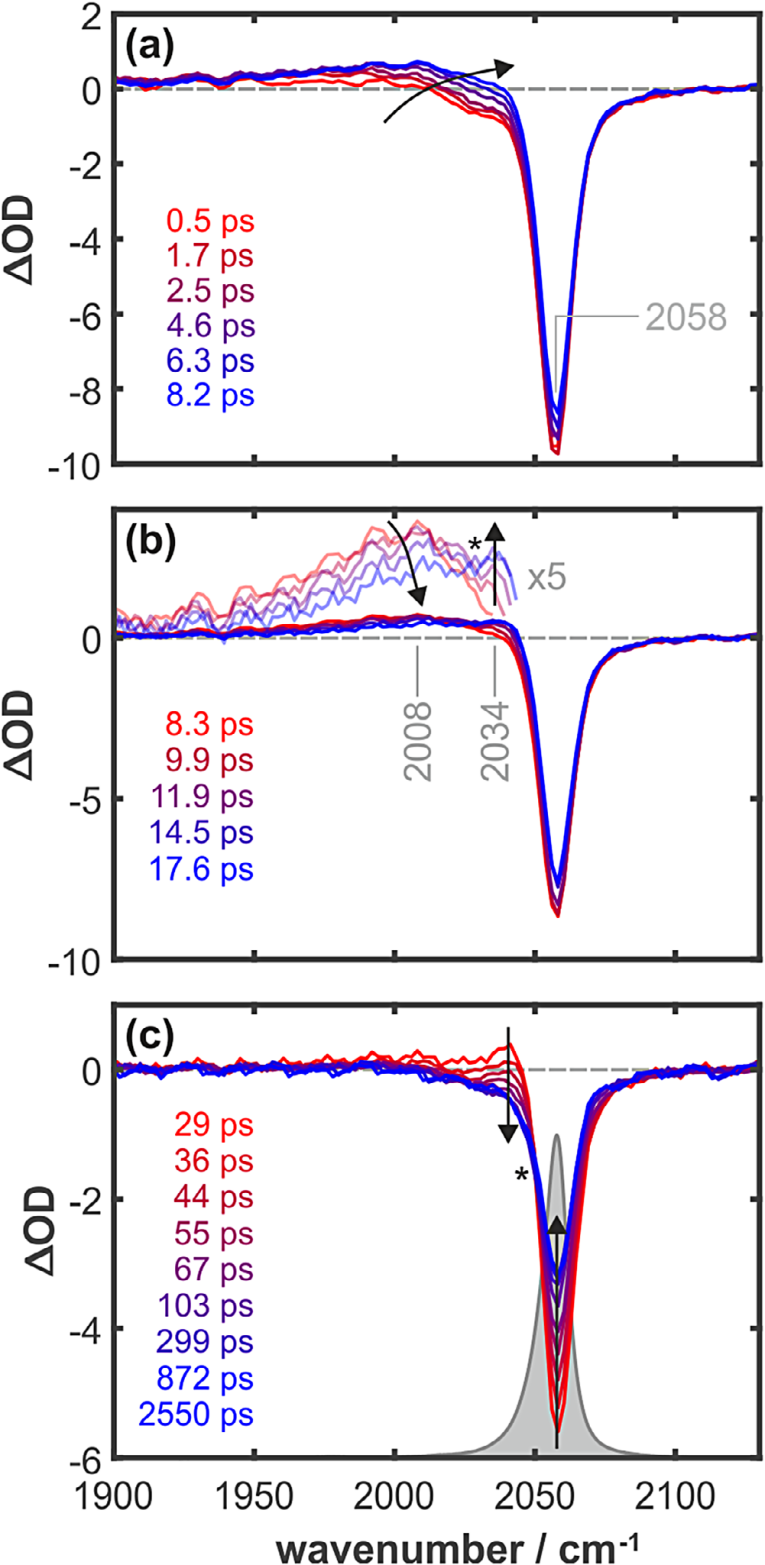
266 nm‐pump/MIR‐probe spectra of **2** in CHCl_3_ solution at room temperature recorded in the azide antisymmetric stretching region for various delays as indicated in the legend. Gray numbers indicate the spectral position in cm^−^^1^. The asterisks highlight isosbestic or near‐isosbestic points. (a) Early delay times between 500 fs and 8.2 ps, (b) intermediate delay times up to 17.6 ps, and (c) later delay times. The FTIR spectrum of **2** is also reproduced at the bottom in panel (c) in gray.

The mid‐infrared (MIR) spectro‐temporal evolution of **2** in the antisymmetric N_3_‐stretching region following ultrafast excitation at 266 nm is displayed in Figure 3 for selected pump‐probe delays. Immediately after photoexcitation (Figure 3a, 0.5 ps), a negative differential optical density (ΔOD) band centered at 2058 cm^−1^ is observed, which spectrally coincides with the maximum of the ground‐state's linear absorption in the same spectral region. This signal arises as a consequence of the pump‐induced population depletion of the electronic ground‐state at zero‐time delay, and thus, must be assigned to ground‐state bleach (GSB). Concurrently, a red‐shifted positive band extending from 1900 to 2016 cm^−^^1^ is also observed at the earliest delay times. Given its prompt appearance, this induced absorption (IA) is attributed to an electronically excited state, 2*. Within only a few picoseconds, the IA band shifts dynamically to higher wavenumbers peaking at 2008 cm^−1^, and at the same time, the GSB band marginally recovers.

Although the extremely broadened IA band in combination with the limited spectral detection window prevents an accurate estimation of the IA‐band integral at 500 fs, a pronounced IA‐deficit relative to the GSB is already apparent. At later delay times, the UV/MIR spectrum recorded at 8 ps yields an IA‐to‐GSB band area ratio of approximately 0.3. The reduced IA‐to‐GSB area ratio suggests that a substantial fraction of the excited‐state population no longer retains the intense azide antisymmetric stretching transition characteristic of the ground state. Under the assumption that the transition dipole strength is approximately conserved for azide‐containing species, the data would correspond to an upper‐limit estimate of ca. 70% conversion into species that are weakly absorbing or silent in this spectral region. This conclusion is supported by our previous findings where coordination of an anionic azido ligand to a transition‐metal center does not significantly alter its antisymmetric stretching transition dipole [12, 51, 52, 53, 54]. In addition, just like free N_3_^●^, azidyl‐radical ligands exhibit exceedingly small IR transition dipoles [54, 55, 56]. These observations are consistent with the azide‐to‐diphenylsulfone LLCT character of the optically pumped excited state (see above). Excitation at 266 nm effectively shifts electron density away from the N_3_ ligand thereby giving it a partial radical character. Although the initially accessed bright states are assigned to LLCT and intra‐ligand excitations, previous studies have shown that such states can rapidly evolve into azide‐centered excited states that are spectroscopically dark in the azide antisymmetric stretching region but decisive for subsequent N─N bond cleavage [12, 13]. From 8 ps onward (see Figure 3b), the area of the IA band decays while a new IA band emerges concomitantly at 2034 cm^−^^1^, giving rise to what appears at first glance to be an isosbestic point at 2026 cm^−^^1^ (cf. the asterisk in Figure 3b). For even longer delays (cf. Figure 3c), the 2034 cm^−^^1^ band fully decays while the GSB partially recovers. These dynamics suggest a relaxation path that recovers the electronic ground state from an azide‐bright excited state absorbing at 2034 cm^−^^1^. Assuming negligible overlap from residual induced absorption at 2058 cm^−^^1^, the persistent bleach corresponds to an approximate upper‐limit estimate of a photochemical conversion yield in the order of 30%.

The observation of a photoproduct that is IR‐silent in the azide antisymmetric stretching region suggests the formation of a nitrene intermediate. To test this hypothesis, we conducted a quenching experiment (see Supporting Information for experimental details) using *t*‐butyl isonitrile (*t*‐BuNC), a well‐established trap for nitrenes that reacts to generate carbodiimides [57]. The asymmetric stretching vibration of the carbodiimide moiety is known to be a very strong IR‐absorber and is expected to appear in the heterocumulenic stretching region between 2100 and 2200 cm^−1^ [54, 58]. Two identical solutions of **2** in the presence of *t*‐BuNC at a molar ratio of 1:50 were prepared: one of them was continuously irradiated with 266 nm femtosecond pulses for 4 h, while the other was kept in the dark as a reference sample. FTIR spectra were recorded at hourly intervals, and differential absorption spectra were obtained by referencing the irradiated solution against the dark control solution. The resulting spectra are shown in Figure 4. After just one hour of irradiation, two negative differential absorption bands appeared at 2058 and 2142 cm^−^^1^, corresponding to the ground‐state FTIR signals of **2** and *t*‐BuNC, respectively, indicating the consumption of both **2** and the quencher. Over the subsequent hours, the negative band at 2058 cm^−^^1^ intensified, while the intensity of the band at 2142 cm^−^^1^ diminished and gave rise to a new positive signal at 2147 cm^−^^1^, tentatively assigned to a carbodiimide‐type product.

**FIGURE 4:**
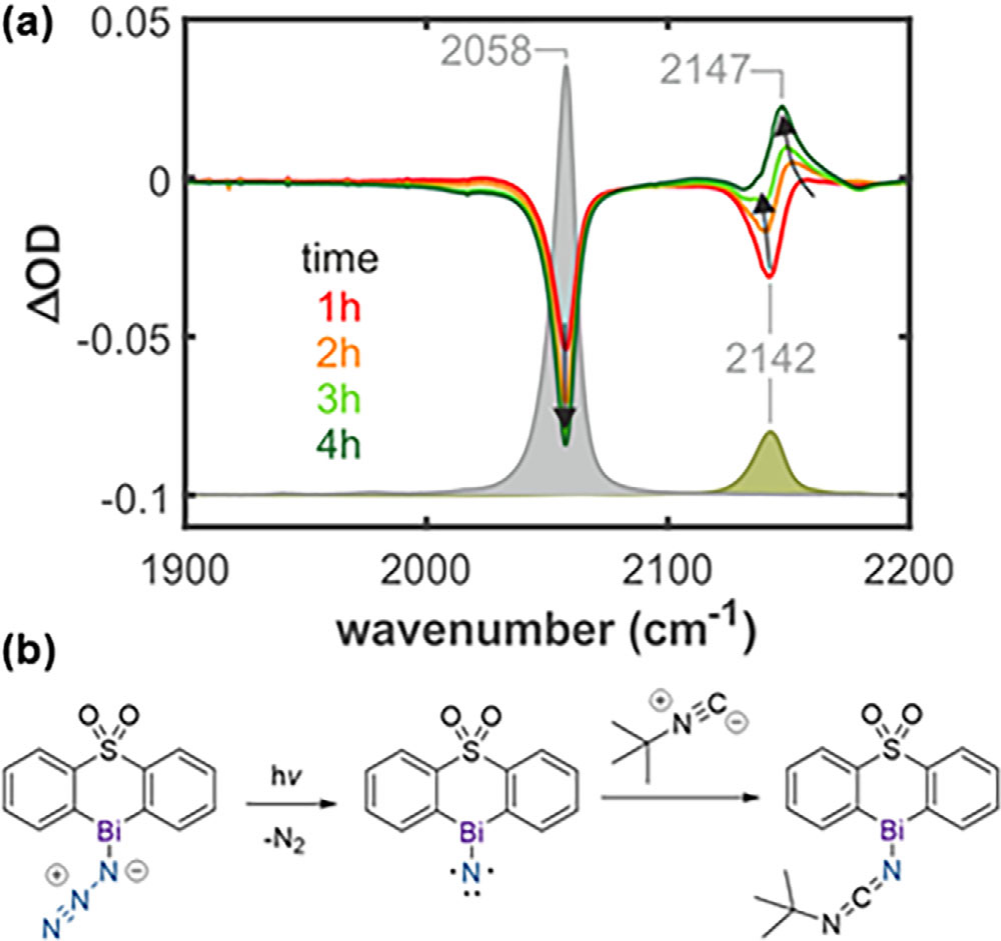
(a) FTIR spectra at hourly intervals following 266 nm‐photolysis of **2** in the presence of *t*‐BuNC in CHCl_3_ at room temperature and (b) scheme of the reaction. Numbers indicate peak positions in cm^−^^1^.

An independent synthesis of the putative carbodiimide product was attempted by irradiating a solution of **2** in CDCl_3_ (0.02 M) containing a 50‐fold excess of *t*‐BuNC in a quartz NMR tube using a mercury vapor lamp (*λ*_max_ = 253 nm) at room temperature. Under these conditions, the experiment yielded a complex mixture of unidentified products. This outcome is not necessarily inconsistent with the transient spectroscopic observations, as the continuous‐wave broadband irradiation conditions used for the preparative photolysis differ substantially from the femtosecond one‐photon excitation regime employed in the pump–probe experiments. In particular, prolonged irradiation increases the likelihood of secondary excitation and subsequent photochemistry of both transient intermediates and primary photoproducts, thereby promoting competing decomposition pathways and product scrambling. An alternative synthetic approach employing triaryl‐ and trialkylphosphines in combination with the azide precursor, followed by quenching with an alkylisocyanate, likewise resulted only in recovery of unreacted starting materials.

Electron paramagnetic resonance (EPR) spectroscopy could in principle provide valuable complementary information regarding the electronic structure of the transient photoproducts, provided that sufficiently long‐lived open‐shell species are formed. However, characterization of nitrene‐type intermediates by EPR is typically performed under cryogenic matrix‐isolation conditions in order to suppress rapid relaxation and secondary reactivity. Such conditions differ fundamentally from the present experiments, which are designed to probe the ultrafast dynamics following one‐photon excitation in fluid solution at room temperature. In the present system, the transient species are generated only under femtosecond irradiation conditions and are not expected to accumulate to concentrations compatible with conventional steady‐state EPR detection. While complementary low‐temperature or time‐resolved EPR studies could provide additional mechanistic insight, such experiments would probe a substantially different photophysical regime and are therefore beyond the scope of the present work.

Alternative mechanistic scenarios, including transient Bi─N_3_ homolysis and azidyl‐radical formation, were considered. Additional trapping experiments employing iodide (I^−^) and 1,1‐dichloroethylene as radical scavengers did not yield distinct new IR absorption features attributable to radical‐derived products under the conditions employed. While these experiments do not exclude transient radical‐type intermediates, they provide no positive evidence for freely diffusing azidyl radicals as the dominant long‐lived photoproduct pathway. Interpretation is further complicated by competing thermal reactivity observed in both control systems. Taken together, the ultrafast MIR experiments reveal rapid and substantial perturbation of the azide electronic structure following 266 nm excitation, including pronounced attenuation of the azide antisymmetric stretching transition and the formation of transient species with strongly altered vibrational signatures. The accompanying trapping experiments with *t*‐BuNC further demonstrate the generation of reactive nitrogen‐centered intermediates capable of producing carbodiimide‐type products. While the present data do not provide unambiguous spectroscopic proof for a discrete bismuth nitrene species, the combined observations are consistent with the formation of nitrene‐like or nitrene‐equivalent intermediates following photoinduced azide activation. These findings identify heavy main‐group azides as promising platforms for exploring photoinduced nitrogen‐transfer chemistry and transient reactive intermediates under mild solution‐phase conditions.

After these observations, we wondered whether a chemical activation of the azide fragment would potentially generate a divergent reactivity. Coordination of organic azides to borane‐based Lewis acids (LAs) is reported to facilitate N_2_ release with the formal production of a nitrene, at which point a substituent of the starting borane migrates on the nitrogen atom to produce an iminoborane derivative [26]. More recently, this reactivity has been re‐enforced by Schulz and co‐workers who reacted HN_3_ and a series of organic azides with BCF and observed both the corresponding Lewis acid–base adduct and the subsequent product of N_2_ release [25]. Inspired by this, we proceeded with the reaction of the azide **2** with 1.0 equivalent of BCF in toluene‐*d_8_*. The ^1^H and ^19^F NMR spectrum showed clean conversion to a new species which was later proved to be the BCF adduct (**3**) via single crystal X‐ray diffraction (Figure 5). The molecular structure revealed a sensitive shortening of the Bi1—O1 distance (2.5328(16) Å) together with an elongation of the Bi1─N1 bond length (2.3964(17) Å) compared to the uncoordinated azide (in **2**: Bi1─O1 = 2.751(3) Å; Bi1─N1 = 2.265(4) Å). The difference in bond lengths between N1─N2 and N2─N3 increases upon BCF coordination, from 0.042 Å in **2** to 0.128 Å in **3** (in **3**: N1─N2 = 1.249(2) Å, N2─N3 = 1.121(3) Å), showing a higher triple bond character between N2 and N3. The Bi1─N1─N2 angle is narrower than the same angle measured in the azide starting material (in **3**: Bi1─N1─N2 = 114.50(13)°; in **2**: 117.1(3)°). The distance between B1 and N1 is shorter for **3** (1.626(3) Å) than that from Schulz and of other organic aides, which average is 1.663 Å. The set of distances B1─N1, N1─N2, and N2─N3 is however very similar to the bond distances reported by Power and coworkers for a tin azide that coordinates BCF, where N1─N2 = 1.244(5) Å, N2─N3 = 1.127(4) Å, and B1─N1 = 1.638(9) Å).

**FIGURE 5:**
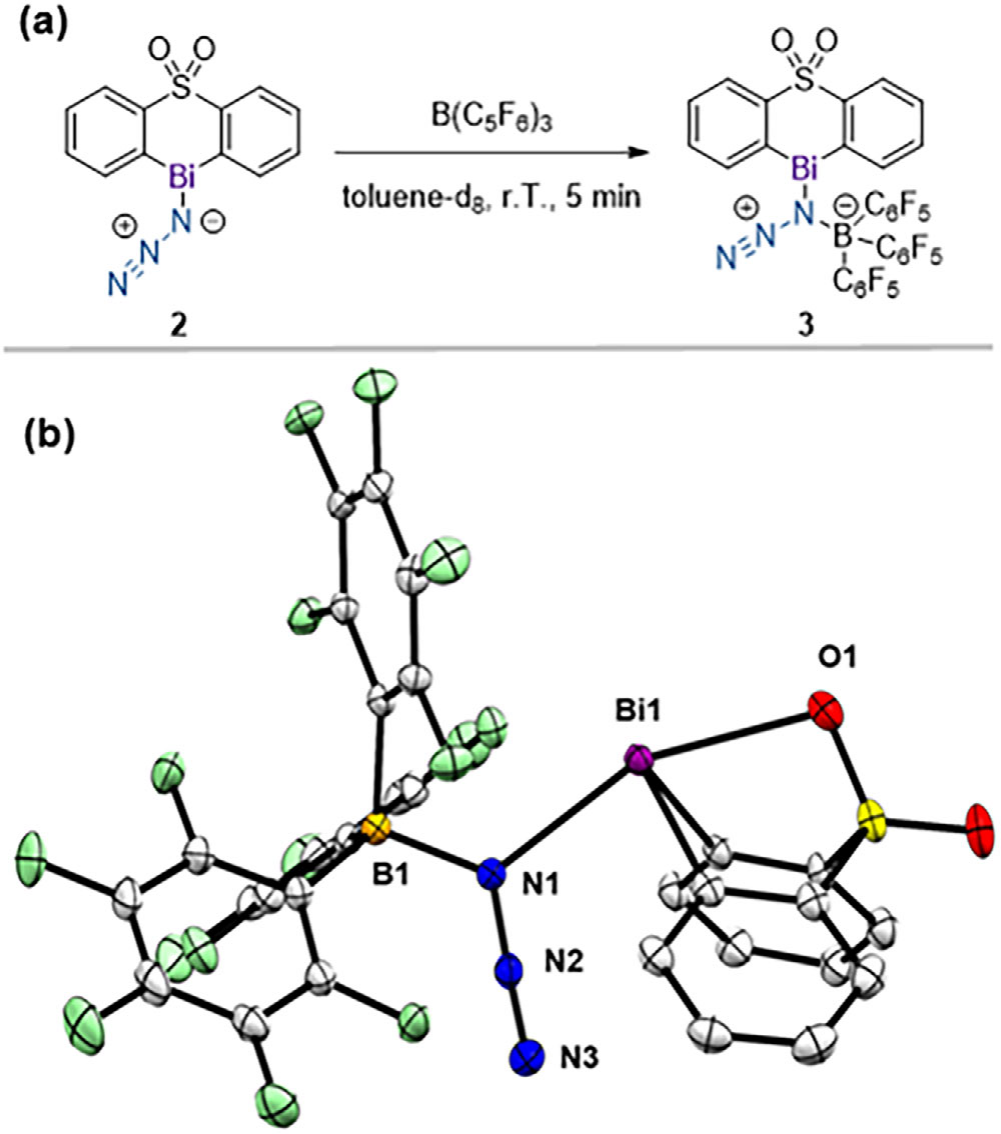
(a) Synthetic route for compound **3**; (b) Single‐crystal x‐ray structure of compound **3**. Thermal ellipsoids are set at 50% probability. Hydrogens omitted for clarity. Selected bond lengths (Å) and angles (°): Bi1─N1 = 2.3964(17); Bi1─O1 = 2.5328(16); N1─N1 = 1.249(2); N2─N3 = 1.121(3); B1─N1 = 1.626(3); Bi1─N1─N2 = 114.50(13).

With the goal of observing nitrene‐like reactivity from our bismacyclic azides, we attempted to expose **3** to UV light irradiation, but it resulted in unselective decomposition of the sample. We then decided to further evaluate the lability of **3** by heating a solution of **3** in toluene‐*d_8_* at 120°C, whereupon we observed the very selective formation of a new species in the ^1^H and ^19^F NMR after 3 days of continuous heating. After leaving the same solution at room temperature overnight, we observed the formation of numerous colorless crystals. X‐ray diffraction of the so obtained crystals revealed formation of an unexpected boracycle (Figures 6, **4a**), which has only one structurally characterized analog present in literature, reported by Braunschweig and coworkers [59]. Compound **4a** shows identical bond lengths parameters for the B1─N2─N3─N4─N5─B2 fragment to the analog (bond lengths respectively for **4a** and Brauschweig´s report: B1─N2 = 1.554(2) Å vs. 1.558(5) Å; B2─N2 = 1.427(2) Å vs. 1.431(4) Å; B2─N5 = 1.444(2) Å vs. 1.439(5) Å; N4─N5 = 1.3484(18) Å vs. 1.355(3) Å; N3─N4 = 1.2763(17) Å vs. 1.284(4) Å; N2─N3 = 1.3669(17) Å vs. 1.367(3) Å). In contrast, the B1─N1 bond length and the N1─B1─N2 angle differ slightly, possibly due to the different substituents on the boron (parameters respectively for **4a** and Brauschweig´s report: B1─N1 = 1.625(2) Å vs. 1.604(3) Å; N1─B1─N2 = 102.80(11)° vs. 106.40°). Examination of the composition of **4a** revealed that the reaction stoichiometry would necessitate the concurrent formation of a bismuth species bearing the missing pentafluorophenyl substituent. To confirm the presence of this species during the pyrolysis of **3**, we independently synthesized pentafluorophenyl phenothiabismine 5,5‐dioxide (**5**) from precursor **1** using lithium pentafluorophenylide (see Supporting Information). The ^1^H and ^19^F NMR spectra of the crude pyrolysis mixture matched the ones of the isolated compound **5**, confirming our hypothesis.

**FIGURE 6:**
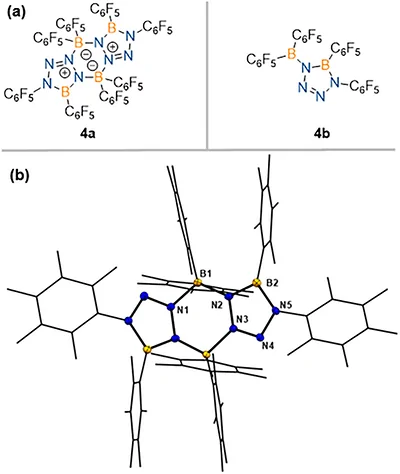
(a) Molecular scheme of **4a**; (b) Single crystal x‐ray structure of **4a**. Thermal ellipsoids are set at 50% probability. Hydrogens omitted for clarity. The ellipsoids for the peripheral aryl groups were omitted for clarity. Selected bond lengths (Å) B1─N2 = 1.554(2) Å; B2─N2 = 1.427(2) Å; B2─N5 = 1.444(2) Å; N4─N5 = 1.3484(18) Å; N3─N4 = 1.2763(17) Å; N2─N3 = 1.3669(17) Å.

Structurally related compounds to **4a** have recently attracted attention in boron chemistry, with several examples reported by Braunschweig and coworkers [59]. It should be noted that although **4a** has been previously reported by Paetzold and coworkers, no structural characterization was given, and that its controlled production via our synthesis instead directly affords the crystalline product and allows an easier workup and overall characterization [60, 61]. Species like **4a** are proposed to arise from dimerization of tetrazaborole monomers (e.g., **4b**) [59]. As suggested by Paetzold and co‐workers, tetrazaboroles are formed via cycloaddition between an aryl‐substituted iminoborane and the corresponding boron azide [60, 61]. In this sequence, a boron–nitrene complex (Ar_2_BN) rearranges to the iminoborane (ArBNAr) through aryl migration from boron to nitrogen, followed by (3+2) cycloaddition with the parent azidoborane to yield the tetrazaborole. This Lewis‐acid‐mediated pathway serves as the chemical counterpart to the photochemical activation manifold; whereas the former is here proposed to yield a transient bismuth‐centered nitrene through N_2_ extrusion, the latter utilizes the bismacycle as a template for boron‐centered transformations, enabling a selective transmetalation‐cycloaddition sequence that significantly expands the reactivity landscape of heavy pnictogen azides.

## 3 Conclusion

In summary, we have introduced a novel bismuth azide featuring a Suzuki‐type ligand framework that exhibits remarkable stability toward air exposure and a wide range of reaction conditions. Ultrafast UV‐pump/MIR‐probe spectroscopy reveals that photoexcitation of compound **2** at 266 nm induces rapid depletion of the azide antisymmetric stretching vibration, accompanied by the emergence of a broad, red‐shifted transient absorption band with significantly reduced intensity. After 3 ns, only the ground‐state bleach associated with the azide antisymmetric stretch remains detectable. Complementary chemical trapping experiments with *t*‐butyl isonitrile, monitored by FTIR spectroscopy, produce a new heterocumulenic stretching absorption, tentatively assigned to a carbodiimide species, consistent with the transient formation of a nitrene intermediate upon photolysis. A detailed mechanistic analysis of the photochemical process is beyond the scope of the present work and will be addressed in a forthcoming dedicated study. In contrast, thermochemical activation proceeds via transmetalation to generate a boron azide and its iminoborane derivative, which undergoes cycloaddition to afford a tetrazaborole dimer. This divergence between photochemical and Lewis‐acid activation highlights the unique mechanistic pathways accessible to heavy pnictogen azides and opens new routes for controlled generation of reactive intermediates. By expanding the reactivity landscape of bismuth azides, this work provides a new, stable platform for exploring heavy‐element nitrene chemistry and suggests broader opportunities for selective activation strategies in main‐group and organometallic synthesis.

## Conflicts of Interest

The authors declare no conflicts of interest.

## Supporting information



The authors have cited additional references within the Supporting Information [62, 63, 64, 65, 66, 67, 68, 69, 70].
**Supporting File**: chem71556‐sup‐0001‐SuppMat.pdf

## Data Availability

The data that supports the findings of this study are available in the supplementary material of this article.
