# Supplementary material for: Photochemical and Thermal Activation of a Diarylbismuth Azide
Source: Chemistry. 2026 Aug 13;32(41):e71556. doi: 10.1002/chem.71556 (PMC13729950; doi:10.1002/chem.71556)
Supplement: Supplementary file 1 [file CHEM-32-e71556-s001.pdf]

Photochemical and Thermal Activation of a Diarylbismuth Azide

Giacomo Pugliese,[a] Oliver Pichl,[b] Gregor Schnakenburg,[a] Peter Vöhringer,[b] Luis I. Domenianni,*[b] Alessandro Bismuto*[a]

[a]*Institute of Inorganic Chemistry, University of Bonn, Gerhard-Domagk-Str. 1, 53121 Bonn, Germany*;

[b]*Clausius-Institut für Physikalische und Theoretische Chemie, University of Bonn, Wegelerstrasse 12, 53115 Bonn*

*ldomenia@uni-bonn.de

*bismuto@uni-bonn.de

Table of Contents

General information ... 3

Synthesis ... 4

Synthesis of 10-(4'-fluorophenyl)phenothiabismine 5,5-dioxide ... 4

Synthesis of 10-iodophenothiabismine 5,5-dioxide (1) ... 5

Synthesis of 10-azidophenothiabismine 5,5-dioxide (2) ... 6

Synthesis of 10-azidophenothiabismine 5,5-dioxide tris(pentafluorophenyl)borane adduct (3) ... 7

Thermolysis of 10-azidophenothiabismine 5,5-dioxide tris(pentafluorophenyl)borane adduct ... 8

Synthesis of 10-(pentafluorophenyl)phenothiabismine 5,5-dioxide ... 9

Spectroscopic analysis ... 10

Experimental ... 10

Computational ... 12

Fourier transform infrared spectra ... 14

NMR spectra ... 15

Single crystal X-ray diffraction analysis ... 24

References ... 27

General information

Unless otherwise specified, all reagents were used as received from commercial suppliers (ABCR, AcrosOrganics, Alfa Aesar, BLDpharm, Sigma Aldrich, Thermo Fisher Scientific). $BiBr_3$ was purified by sublimation under reduced pressure and stored under Ar atmosphere prior to use. ^{1}H and $^{13}C\{^{1}H\}$ NMR spectra were recorded in toluene-d_8 or $CDCl_3$ at 298 K (otherwise specified) using a Bruker Avance I 300 MHz, Bruker Avance I 400 MHz, and Bruker Avance III HD Ascend 500 MHz. ^{1}H NMR was reported as follows: chemical shift, multiplicity (s = singlet, d = doublet, and t = triplet), coupling constant (J values) in Hz and integration. Chemical shifts (δ) were reported with respect to the corresponding solvent residual peak at 2.09 ppm for toluene-d_8, and 7.26 ppm for $CDCl_3$. ^{13}C NMR spectra (^{1}H broadband decoupled) were reported in ppm using the central peak of $CDCl_3$ (77.2 ppm) or the -CD_3 peak of toluene-d_8 (20.4 ppm). Tri(4-fluorophenyl)bismuthine was synthesized according to literature.[1] Unless otherwise specified, the syntheses were carried out in a glovebox (GS MEGA 3) under an argon atmosphere or using standard Schlenk techniques. Tetrahydrofuran (THF), *n*-hexane, and *n*-pentane were dried over sodium, distilled and stored over molecular sieves (3 Å) prior to use.

Synthesis

Synthesis of 10-(4'-fluorophenyl)phenothiabismine 5,5-dioxide

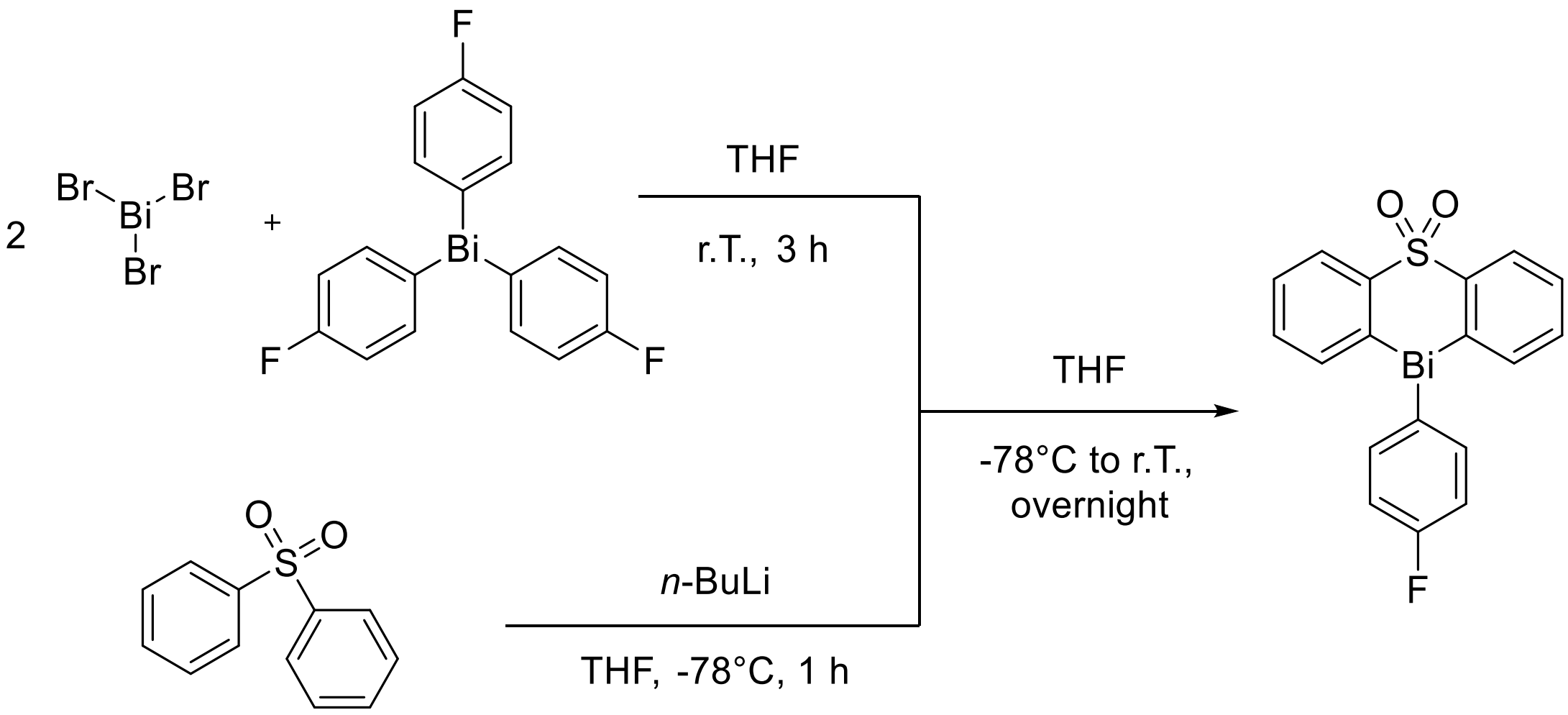

$BiBr_3$ (1.803 g, 4.02 mmol, 0.66 equiv.) and tris(4-fluorophenyl)bismuthane (1.013 g, 2.05 mmol, 0.34 equiv.) were added to a Schlenk tube and dissolved in dry THF (20.0 mL), and the solution was left stirring at room temperature covered in aluminium foil for 3 h, thereby producing 4-fluorophenylbismuth dibromide. Meanwhile, a solution of the lithiated ligand was prepared by adding *n*-BuLi (2.5 M in *n*-hexane, 11.0 mL, 27.50 mmol, 2.18 equiv.) to a stirred solution of diphenylsulfone (2.672 g, 12.24 mmol, 1.05 equiv.) in dry THF (20.0 mL) at -78°C, whereupon a pale-yellow precipitate formed. The suspension was left stirring at -78°C for 1 h and 4-fluorophenylbismuth dibromide was then slowly added via cannula at -78°C. After the addition was completed, the so obtained clear dark yellow solution was allowed to warm up to room temperature and left stirring at the same temperature overnight. The reaction was quenched with sat. NH_4Cl (aq) for 5 minutes and the aqueous phase was extracted with EtOAc (2 x 40.0 mL) and DCM (2 x 40.0 mL). The collected organic fractions were dried over Na_2SO_4 and filtered through cotton. The solvent was then removed under reduced pressure and the solid residue was purified by column chromatography (*n*-hexane:EtOAC 4:1) to afford the product as a white crystalline solid (4.743 g, 76%).

^{1}H NMR (500 MHz, $CDCl_3$) δ 8.38 (dd, *J* = 7.6, 1.4 Hz, 2H), 7.85 (dd, *J* = 7.3, 1.3 Hz, 2H), 7.75 – 7.68 (m, 2H), 7.41 (td, *J* = 7.6, 1.3 Hz, 2H), 7.36 (td, *J* = 7.3, 1.4 Hz, 2H), 7.11 – 7.03 (m, 2H).

^{13}C{H} NMR (126 MHz, $CDCl_3$) δ 163.2 (d, *J* = 248.4 Hz), 160.7, 158.8, 141.9, 141.0 (d, *J* = 7.2 Hz), 137.6, 133.7, 128.5, 127.3, 118.4 (d, *J* = 19.8 Hz).

^{19}F{H} NMR (470 MHz, $CDCl_3$) δ -111.18 (tt, *J* = 9.3, 6.1 Hz).

EA calcd. for $C_{18}H_{12}BiFO_2S$: C 41.55, H 2.32, S 6.16; Found: C 41.60, H 2.43, S 6.44.

HRMS calcd. for $C_{18}H_{12}BiFO_2SH^+$: 521.0424; Found: 521.0419 (ESI^+).

Synthesis of 10-iodophenothiabismine 5,5-dioxide (1)

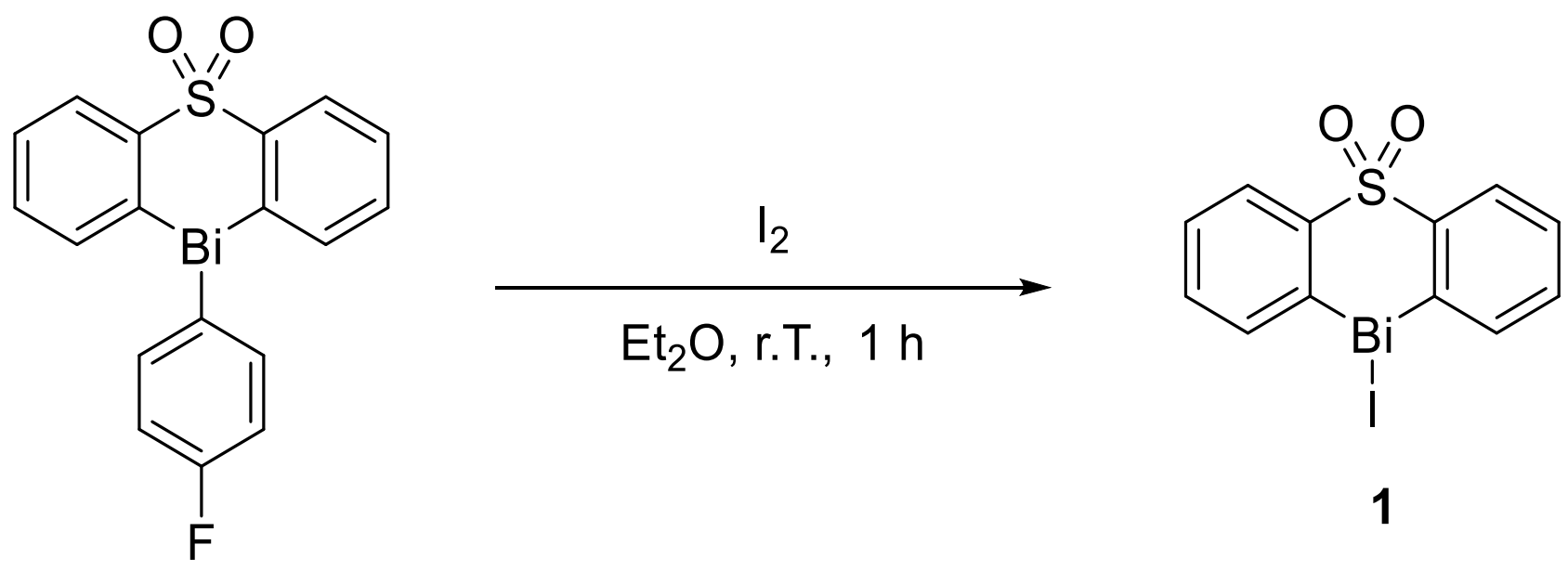

1

The synthesis of 1 was carried using a modified literature protocol.[2] Under ambient conditions, a solution of 10-(4'-fluorophenyl)phenothiabismine (520 mg, 1.00 mmol, 1.00 equiv.) in Et_2O (5.0 mL) was treated with a solution of I_2 (270 mg, 1.06 mmol, 1.06 equiv.) in Et_2O (5.0 mL) added in small portions at room temperature, producing a grey precipitate which was left stirring at room temperature for 1 h. The suspension was then filtered over a P4 frit and the solid was washed twice with Et_2O. The product was obtained after recrystallization from DCM *n*-hexane mixture (1:5 v/v) as a yellow crystalline solid. Single-crystals suitable for X-ray analysis were grown by slow evaporation of a saturated DCM solution.

^{1}H NMR (500 MHz, $CDCl_3$) δ 9.22 (d, *J* = 7.4 Hz, 2H), 8.30 (d, *J* = 7.6 Hz, 2H), 7.61 (d, *J* = 7.5 Hz, 2H), 7.49 (t, *J* = 7.5 Hz, 2H).

^{13}C{H} NMR (126 MHz, $CDCl_3$) δ 163.8, 140.4, 140.3, 136.0, 128.9, 127.7.

EA calcd. for $C_{12}H_8BiIO_2S$: C 26.10, H 1.46, S 5.81; Found: C 26.30, H 1.52, S 5.92.

HRMS calcd. for $C_{12}H_8BiIO_2S$: 552.9172; Found: 552.9166 (ESI^+).

Synthesis of 10-azidophenothiabismine 5,5-dioxide (2)

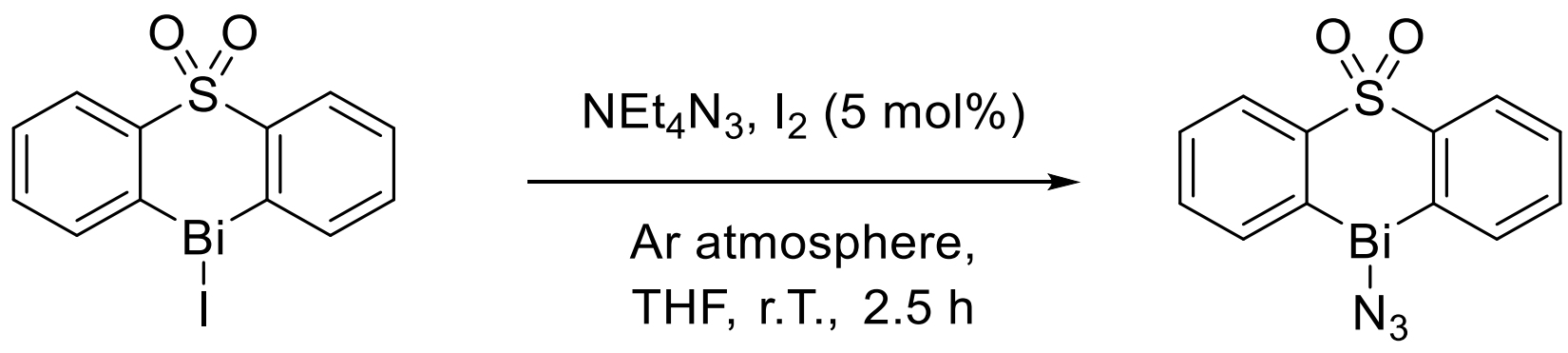

2

10-iodophenothiabismine 5,5-dioxide (55.0 mg, 0.10 mmol, 1.00 equiv.) and tetraethylammonium azide (18.9 mg, 0.11 mmol, 1.10 equiv.) were introduced under inert atmosphere in a vial, followed by 1.0 mL of an I_2 solution (0.05 M) in dry THF. The so obtained brown-red suspension was left stirring vigorously at room temperature for 2.5 h, whereupon the solution decoloured. The suspension was then filtered over glass fibre and the liquid was added under stirring to 10.0 mL of distilled H_2O in a round bottom flask, producing a white precipitate. The solid was collected by filtering the suspension through a P4 frit and washed twice with distilled H_2O. The product was then dried under reduced pressure at 40°C and recrystalized from DCM *n*-pentane mixture (1:5 v/v) giving a white powder (367 mg, 79%). Single-crystal suitable for X-ray analysis were grown from a saturated solution of DCM:*i*-PrOH (2:1 v/v) at −18°C.

^{1}H NMR (500 MHz, $CDCl_3$) δ 8.54 (d, *J* = 7.3 Hz, 2H), 8.40 (d, *J* = 7.7 Hz, 2H), 7.76 (d, *J* = 7.3 Hz, 2H), 7.50 (t, *J* = 7.6 Hz, 2H).

^{13}C{H} NMR (126 MHz, $CDCl_3$) δ 177.6, 140.7, 135.6, 135.0, 129.3, 129.3.

m.p. /°C: 242 (dec.)

EA calcd. for $C_{12}H_8BiN_3O_2S$: N 8.99, C 30.85, H 1.73, S 6.86; Found: N 8.81, C 31.25, H 1.81, 7.20.

HRMS calcd. for $C_{12}H_8BiO_2S$: 425.0049; Found: 425.0051 (ESI$^+$).

Exposure of **2** to blue (λ = 451 nm) or UV (λ = 367 nm) LED light did not have any selective outcome in any of the solvents used (toluene-d_8, CD_2Cl_2, C_6D_6, CD_3CN). As final effort, we irradiated the sample in the solid state at λ = 367 nm for 2 days, but no selective product formation was observed.

These optimized conditions were obtained after a careful evaluation of the equivalents of iodine using the same protocol as above.

Table S1

Entry	Iodine equiv.	Yield (%)
1	1.00	30
2	0.50	45
3	0.25	60
4	0.05	79

Synthesis of 10-azidophenothiabismine 5,5-dioxide tris(pentafluorophenyl)borane adduct (3)

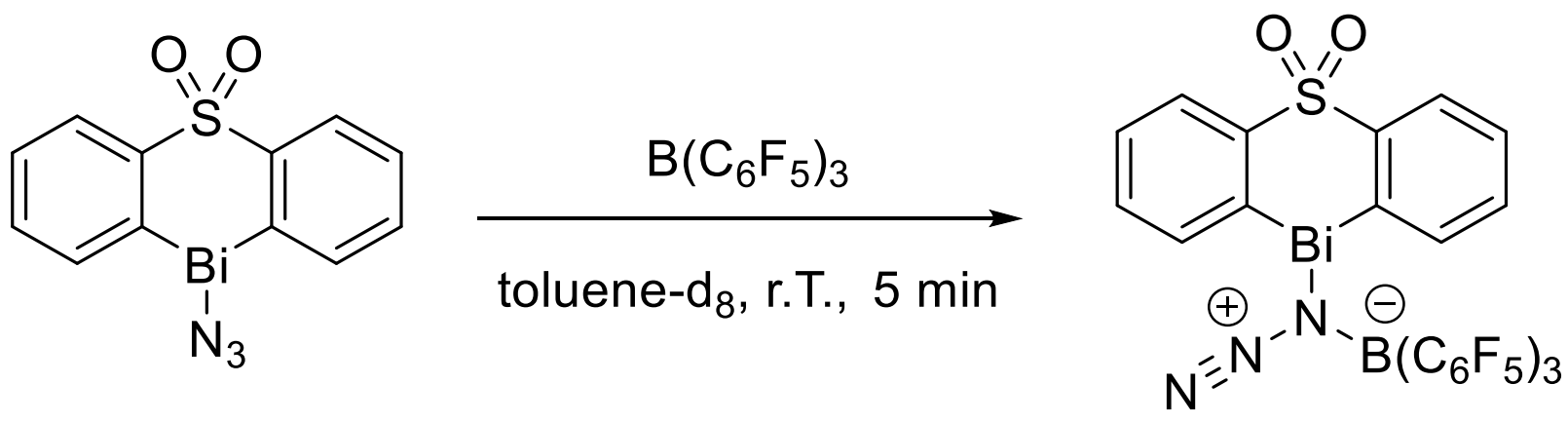

3

A suspension of 10-azidophenothiabismine 5,5-dioxide (4.7 mg, 0.01 mmol, 1.00 equiv.) in 0.3 mL of dry toluene-d_8 was quickly treated with a suspension of tris(pentafluorophenyl)borane (5.1 mg, 0.01 mmol, 1.00 equiv.) in 0.3 mL of dry toluene-d_8. The suspension became clear after mixing for 5 min at r.T.. The solvent was removed under vacuum to afford the product as a white powder in quantitative yield. Single-crystal suitable for X-ray analysis were grown by diffusion of *n*-hexane into a saturated toluene solution at –30°C.

^{1}H NMR (500 MHz, Tol) δ 8.45 (d, *J* = 7.4 Hz, 1H), 7.99 (dd, *J* = 7.7, 1.3 Hz, 1H), 7.08 (td, *J* = 7.8, 1.6 Hz, 1H), 6.65 (td, *J* = 7.6, 1.1 Hz, 1H).

^{13}C{H} NMR (126 MHz, Tol) δ 187.46, 148.39 (d, *J* = 240.4 Hz), 140.8 (d, *J* = 250.2 Hz), 139.73, 137.8 (d, J = 247.4 Hz), 137.05, 133.94, 130.28, 129.77.

^{19}F{H} NMR (470 MHz, Tol) δ –132.87 (dd, *J* = 23.6, 7.9 Hz), –155.32 (t, *J* = 20.7 Hz), –162.01 – 162.56 (m).

^{11}B NMR (160 MHz, Tol) δ – 4.16.

EA calcd. for $C_{30}H_8BBiF_{15}N_3O_2S$: N 4.29, C 36.80, H 0.82, S 3.27; Found: N 4.10, C 37.05, H 0.78, S 3.12.

Thermolysis of 10-azidophenothiabismine 5,5-dioxide tris(pentafluorophenyl)borane adduct

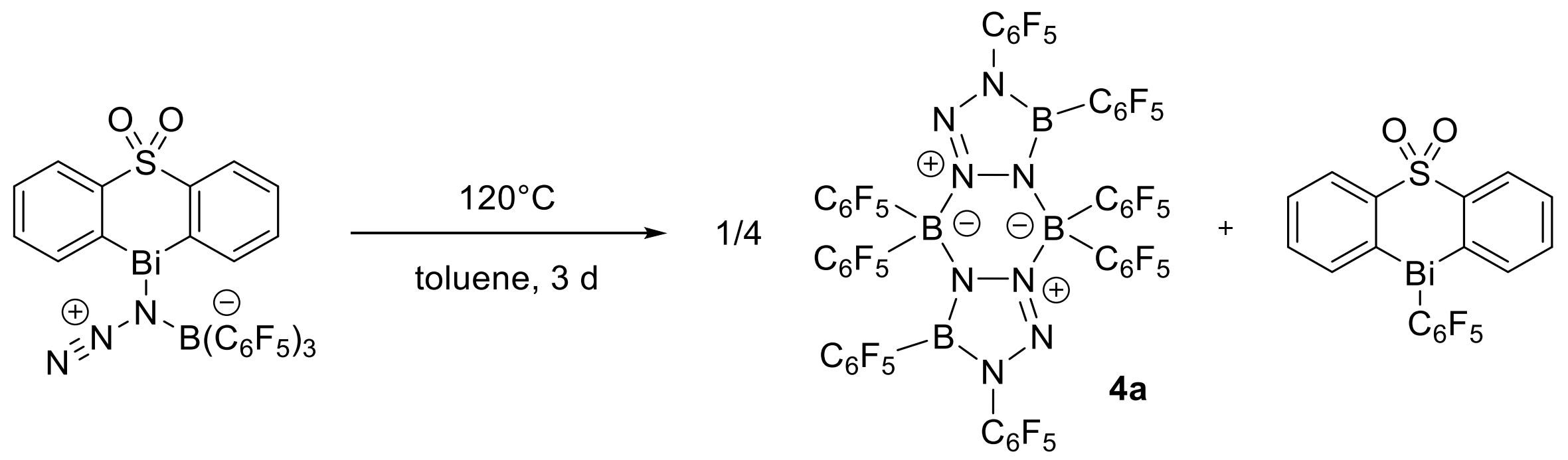

A solution of 10-azidophenothiabismine 5,5-dioxide tris(pentafluorophenyl)borane adduct (9.8 mg, 0.01 mmol) in dry toluene (0.5 mL) was heated in a sealed NMR tube at 120°C for 3 days, whereupon colourless crystals of **4a** eventually formed. After separation from the mother liquor and washing with dry toluene (2 x 0.5 mL) the product was obtained as colourless crystals (3.5 mg, 94%). Single-crystal suitable for X-ray analysis were grown from a saturated toluene solution at –30°C.

Charachterization data for **4a**:

^{1}H NMR no signals.

^{13}C{H} NMR due to the low solubility of **4a**, no signals on the ^{13}C NMR could be observed.

^{19}F{H} NMR (470 MHz, $C_6H_4F_2$) δ –108.94 (m), –114.48 (dd, *J* = 9.9, 4.9 Hz), –116.56 (d, *J* = 18.4 Hz), –116.99 (m), –129.25 (br), –140.24 (d, *J* = 235.7 Hz), –147.16 (t, *J* = 19.0 Hz), –148.83 (d, *J* = 19.0 Hz), – 49.93 (t, *J* = 20.4 Hz), –152.70, –160.62 (ddt, *J* = 37.4, 28.7, 10.3 Hz), –163.50.

^{11}B NMR (128 MHz, $C_6H_4F_2$) δ 27.6, –0.19.

EA calcd. for $C_{48}B_4F_{40}N_8$: N 7.51, C 38.65; Found: N 7.62, C 38.91.

Note: **4a** proved to be insoluble in all of the non coordinating solvents used for HRMS analysis.

Synthesis of 10-(pentafluorophenyl)phenothiabismine 5,5-dioxide (5)

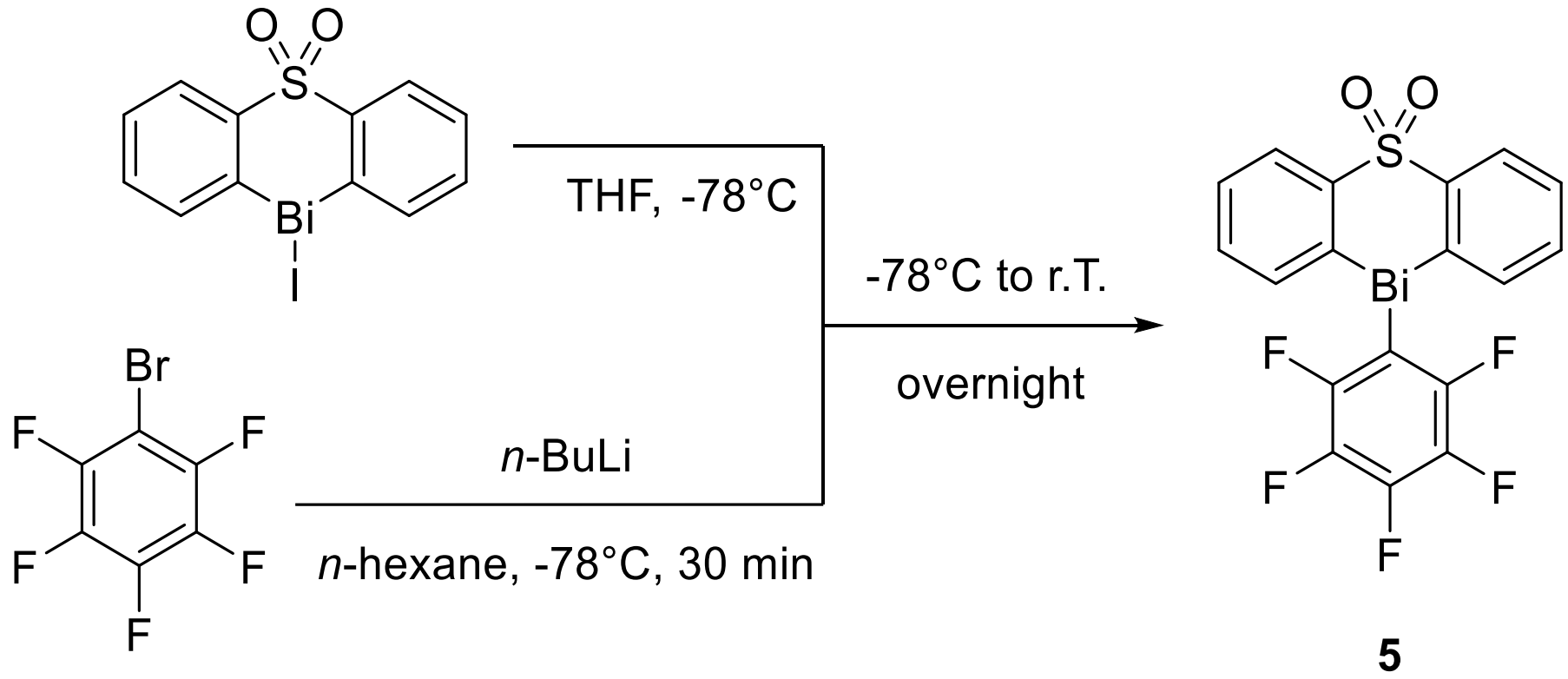

5

A solution of bromo pentafluorobenzene (125 μL, 1.00 mmol, 1.00 equiv.) in dry *n*-hexane (10.0 mL) was treated with *n*-BuLi (2.5 M in *n*-hexane, 0.4 mL, 1.00 mmol, 1.00 equiv.) at -78°C, whereupon a white precipitate formed. 10-iodophenothiabismine 5,5-dioxide (552 mg, 1.00 mmol, 1.00 equiv.) was dissolved in dry THF (10.0 mL) and added dropwise at -78°C to the lithiated ligand. After the addition, the solution was allowed to warm up to r.T. and it was left stirring overnight at that temperature. The reaction was quenched with saturated NH_4Cl(aq), the phases were separated and the aqueous phase was extracted with EtOAc (3 x 30 mL). The combined organic phases were dried over Na_2SO_4, filtered through cotton and concentrated on the rotavapor. The product was purified by recrystallization from hot toluene and obtained as a white powder (240 mg, 41%).

^{1}H NMR (500 MHz, $CDCl_3$) δ 8.45 – 8.40 (m, 2H), 8.19 – 8.11 (m, 2H), 7.51 – 7.39 (m, 4H).

^{13}C{H} NMR (126 MHz, $CDCl_3$) δ 158.87, 149.62 – 146.51 (m), 143.5 – 141.0 (m), 141.34, 139.3 – 136.8 (m), 137.48, 134.09, 128.91, 127.97.

^{19}F{H} NMR (470 MHz, $CDCl_3$) δ –114.90 (m), –150.43 (m), –158.36 (m).

HRMS calcd. for $C_{18}H_8BiF_5O_2S$: 591.9969; Found: 591.9962 (ESI$^+$)

EA calcd. for $C_{18}H_8BiF_5O_2S$: C 36.50, H 1.36, S 5.41; Found: C 36.42, H 1.41, S 5.31.

Spectroscopic analysis

Experimental

Fourier transform infrared (FTIR) spectra were collected with a Bruker Vertex 70 spectrometer at a wavenumber resolution of 0.5 cm^{-1}. Linear electronic absorption spectra in the ultraviolet-visible region (UV-Vis) were taken with a PerkinElmer Lambda 365+ spectrophotometer at a wavelength resolution of 1 nm.

Femtosecond time-resolved UV-pump/MIR-probe measurements were collected with a home-built spectrometer. Briefly, two Ti:sapphire regenerative amplifier front ends (RGA, Solstice-Ace, Newport/Spectra-Physics) were synchronously seeded by a single modelocked Ti:sapphire oscillator (MaiTai, Newport Spectra Physics). A fraction of the 800 nm fundamental output of one of the amplifiers is frequency-tripled to 266 nm for later use as the pump pulse. The pump power was maintained below 6 µJ. A chopper disc blocks every other pump pulse. Using a fused silica lens (f = 400 mm) the UV-beam diameter was reduced to ~1 mm at the sample position where it was spatially overlapped with the detection pulses at a crossing angle of 5°. A fraction of the second RGA's output was used to drive an optical parametric amplifier (TOPAS Prime, Light Conversion) at a pump energy of 350 µJ. The signal and idler pulses of this OPA were frequency-down converted to the MIR-region via difference-frequency generation in a type-I $AgGaS_2$-crystal to obtain probe pulses in the MIR-Region with an energy of up to 10 µJ. The probe-pulses were sent through a Ge-filter to suppress residual signal/idler light and were subsequently split into detection and reference pulses of roughly equal intensities. To introduce relative pump-probe delays up to 8 ns, the MIR-pulses were sent four times through an Ag-coated hollow roof retroreflector mounted on a motorized translation stage (Newport DLS 325, MKS Industries). An Au-coated 90° off-axis parabola (OAP, 10 cm effective focal length) was used to focus the MIR-pulse pairs into the sample where the MIR-beam waist was ca. 400 µm. The relative pump-probe polarization was set to the magic angle to suppress signal contribution from molecular rotations. Behind the sample, another identical OAP re-collimated the MIR pulses, which were then steered separately onto the two entrance slits of an imaging spectrometer (iHR 320, Horiba). A referenced detection of the pump-induced absorbance was enabled with a 2 x 32 pixel HgCdTe-array detector (Infrared associates) attached to one of the spectrometer's exits. The sample solution was circulated with a gear pump (flow rate ~100 mL/min) through a home-built flow cell equipped with two CaF_2 windows whose distance was set to 100 µm with a Pb-spacer. The pump-probe setup was purged with dry air to avoid atmospheric absorptions on the probe pulses.

Quenching Experiments

a) ***tert*-butyl isonitrile quenching**

The quenching experiment was performed under identical illumination conditions to those used in the pump–probe measurements. A QS fluorescence cuvette with an optical path length of 10 mm (in both directions), equipped with magnetic stirring, was used. A solution of **2** (8.6 mM) in $CHCl_3$, containing *tert*-butyl isonitrile as a quencher in a 50:1 molar ratio relative to the compound, was prepared and divided into two identical, sealed portions. One sample was kept in the dark, while the other was subjected to pulsed illumination (1 kHz) for a total of 4 h. During the experiment, the cuvette was rotated by 90° every hour to ensure uniform exposure. Aliquots were withdrawn every hour for FTIR analysis. Differential optical densities at each time point were calculated relative to the corresponding unilluminated sample to account for any thermally induced changes. The isonitrile quencher exhibits a negligible absorption cross section under the applied illumination conditions; therefore, corrections for any optically induced reactivity were not required. All experiments were conducted at room temperature.

b) **1,1-dichloroethylene quenching**

The experiment was carried out following the same general procedure described for the *tert*-butyl isonitrile quenching studies. A $CHCl_3$ solution of 2 (8.3 mM), containing 1,1-dichloroethylene in a 10:1 molar excess relative to the compound, was transferred into a sealed QS fluorescence cuvette (10 mm optical path length) equipped with magnetic stirring. The solution was exposed to pulsed irradiation (1 kHz) for 2h under the same illumination conditions used for the pump–probe measurements. To ensure homogeneous irradiation, the cuvette orientation was rotated by 90° after 1h of illumination. A second identical sample was maintained in the dark. Samples for FTIR analysis were collected at hourly intervals. Spectral changes were evaluated as differential optical densities relative to the corresponding non-irradiated reference sample, thereby minimizing contributions from thermal effects. Owing to the negligible absorption of the quencher at the excitation wavelength, no correction for direct photochemical activation of the quencher was necessary. All measurements were performed at ambient temperature.

c) **tetra-*n*-butylammonium iodide quenching**

A solution of **2** (3.1 mM) in $CHCl_3$ containing tetra-*n*-butylammonium iodide as a quencher in a 10:1 molar ratio relative to the substrate was prepared. To monitor potential background thermal reactivity or decomposition, a control aliquot of this mixture was

loaded into a sealed flow cell (optical path length d = 100 μm, as described in Section UVMIR) and maintained in the dark at ambient temperature; FTIR spectra of this control sample were recorded at 30-minute intervals. The remainder of the solution was reserved for time-resolved rapid-scan FTIR measurements in an identical flow cell and kept in the dark prior to photoexcitation. Rapid-scan FTIR spectra were recorded using a Bruker Vertex 70 spectrometer. Transient species were generated via laser flash photolysis using single excitation pulses (15 mJ, 5 ns pulse width) at a center wavelength of 266 nm, obtained by frequency-quadrupling the fundamental output of a Q-switched Nd:YAG laser (Continuum Surelite II-10). Data acquisition was performed with a time resolution of Δt = 62-67 ms up to a maximum delay time of 50-54 s. A total of 8 experimental runs were collected and averaged over the course of 1 hour to ensure reproducibility.

Computational

Quantum-chemical calculations were carried out within the density functional theory (DFT) and time-dependent (TD) DFT frameworks using the Gaussian 16 program package.[3] TD-DFT calculations were conducted in the Tamm-Dancoff-approximation using the PBE0 (PBE1PBE) hybrid exchange-correlation functional in conjunction with Grimme's D3 dispersion correction with Becke-Johnson damping (D3(BJ)).[4] The def2-TZVP basis set was employed for all atoms. For bismuth, the quasi-relativistic Stuttgart/Cologne effective core potential (ECP) was used, replacing 60 core electrons, together with the corresponding def2-TZVP valence basis set. All geometry optimizations were carried out using Gaussian's "Tight" convergence criteria, an ultrafine integration grid, and without symmetry constraints. Self-consistent field (SCF) convergence was ensured by allowing up to 512 SCF cycles. Relaxed structures were confirmed to be true minima of the potential surface by means of a harmonic normal mode analysis with analytical force constants and verifying the absence of imaginary eigenvalues of the Hessian. A uniform shift of +0.21 eV (~1700 cm^{-1}) was applied to the calculated excitation energies to align the theoretical spectrum with the experimental absorption maxima.

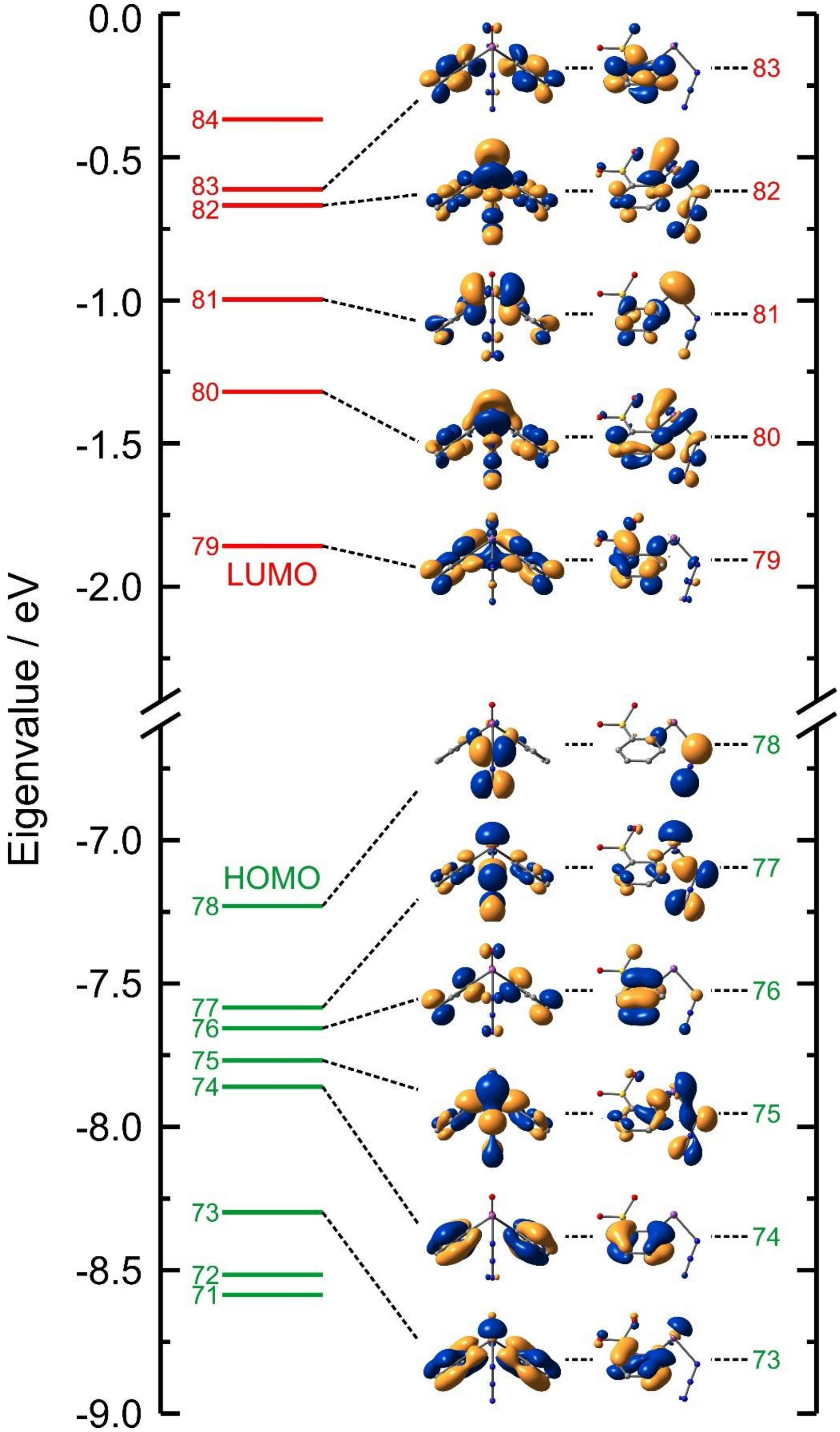

Figure S1. Energies and isosurfaces of the Kohn-Sham molecular orbitals of the singlet electronic ground state of **2** at the PBE0-D3(BJ)/def2-TZVP/CPCM level of theory. Occupied orbitals in green, virtual orbitals in red. Numerals indicate orbital number in the order of increasing energy. The highest occupied molecular orbital (HOMO) is orbital number 78, while the lowest unoccupied molecular orbital (LUMO) is orbital number 79.

Fourier transform infrared spectra

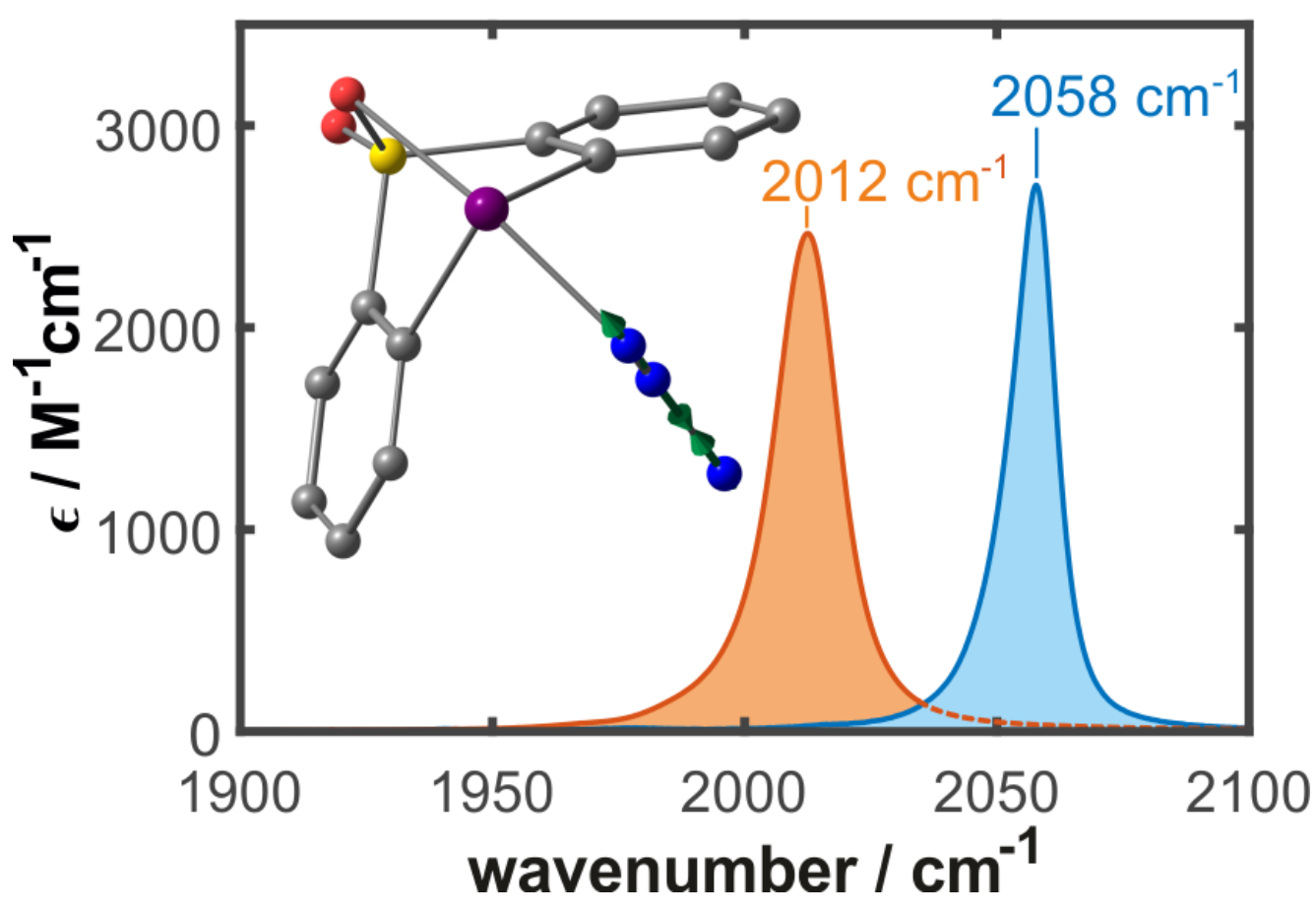

Figure S2: Stationary FTIR spectra of **2** (blue) and NBu_4N_3(orange) measured in $CHCl_3$ solution at room temperature in the antisymmetric N_3-stretching vibration region.

NMR spectra

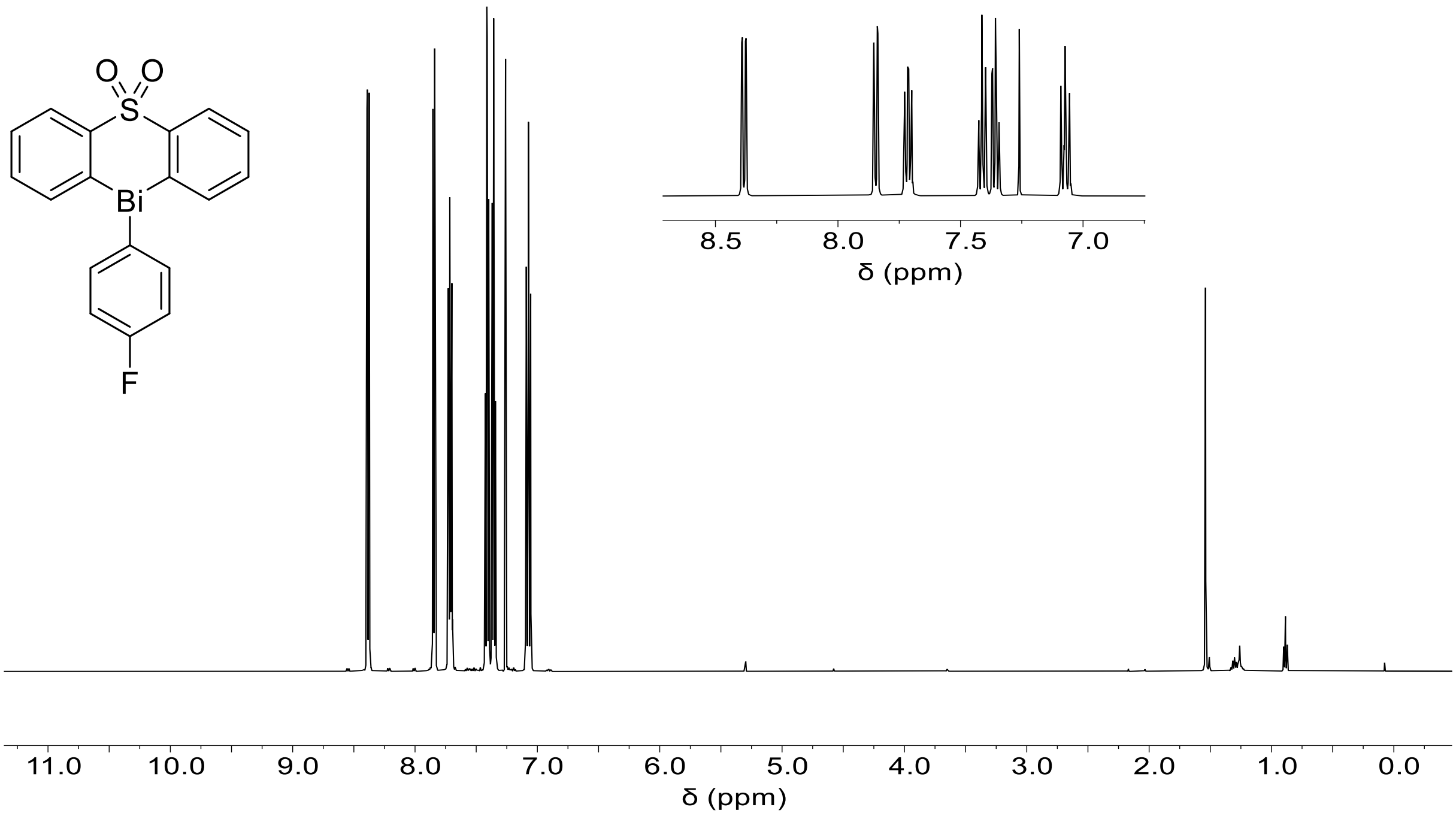

[1]**H NMR** (500 MHz) spectrum of **10-(4'-fluorophenyl)phenothiabismine 5,5-dioxide** in $CDCl_3$.

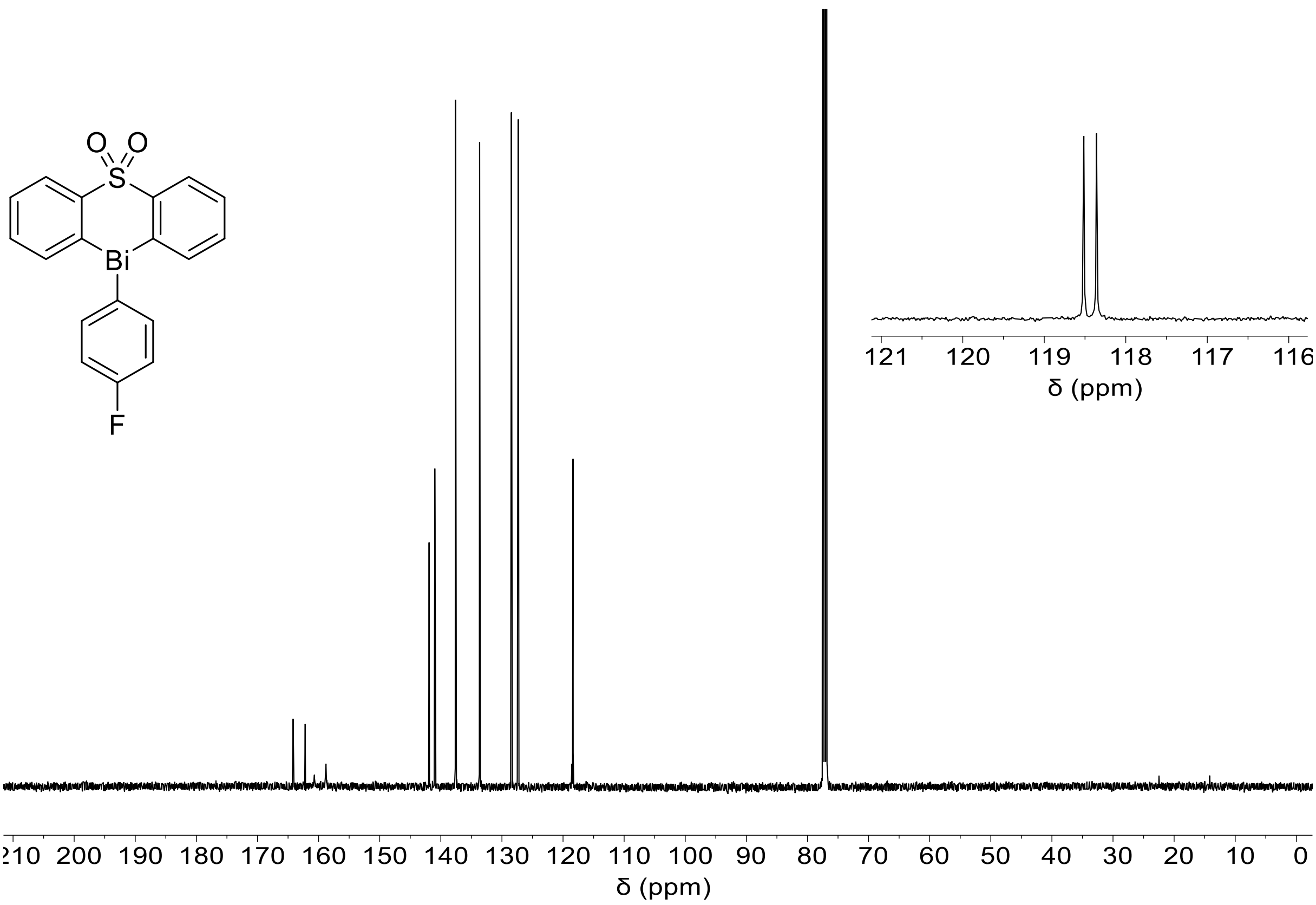

[13]**C{H} NMR** (126 MHz) spectrum of **10-(4'-fluorophenyl)phenothiabismine 5,5-dioxide** in $CDCl_3$.

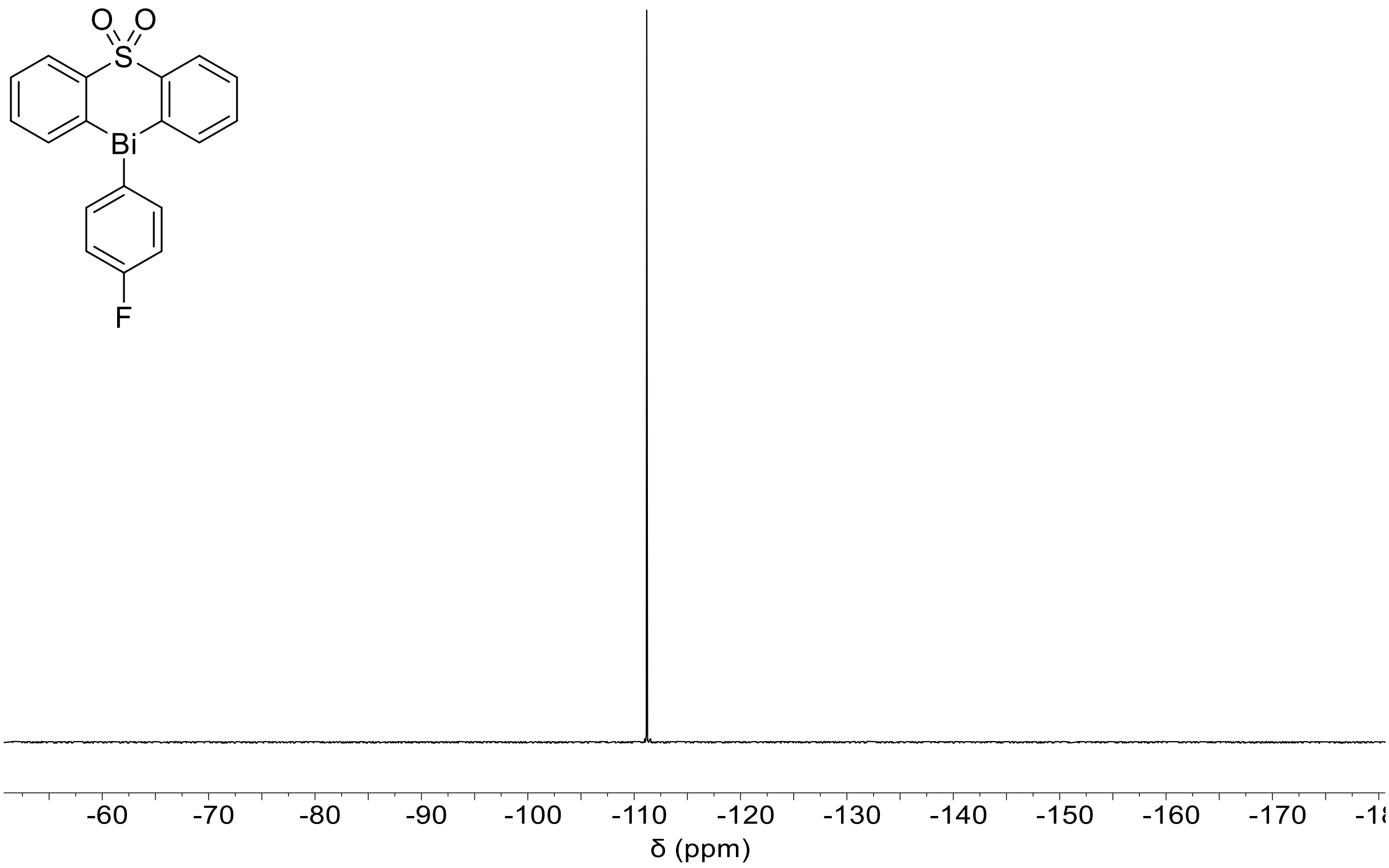

^{19}F NMR (470 MHz) spectrum of **10-(4'-fluorophenyl)phenothiabismine 5,5-dioxide** in $CDCl_3$.

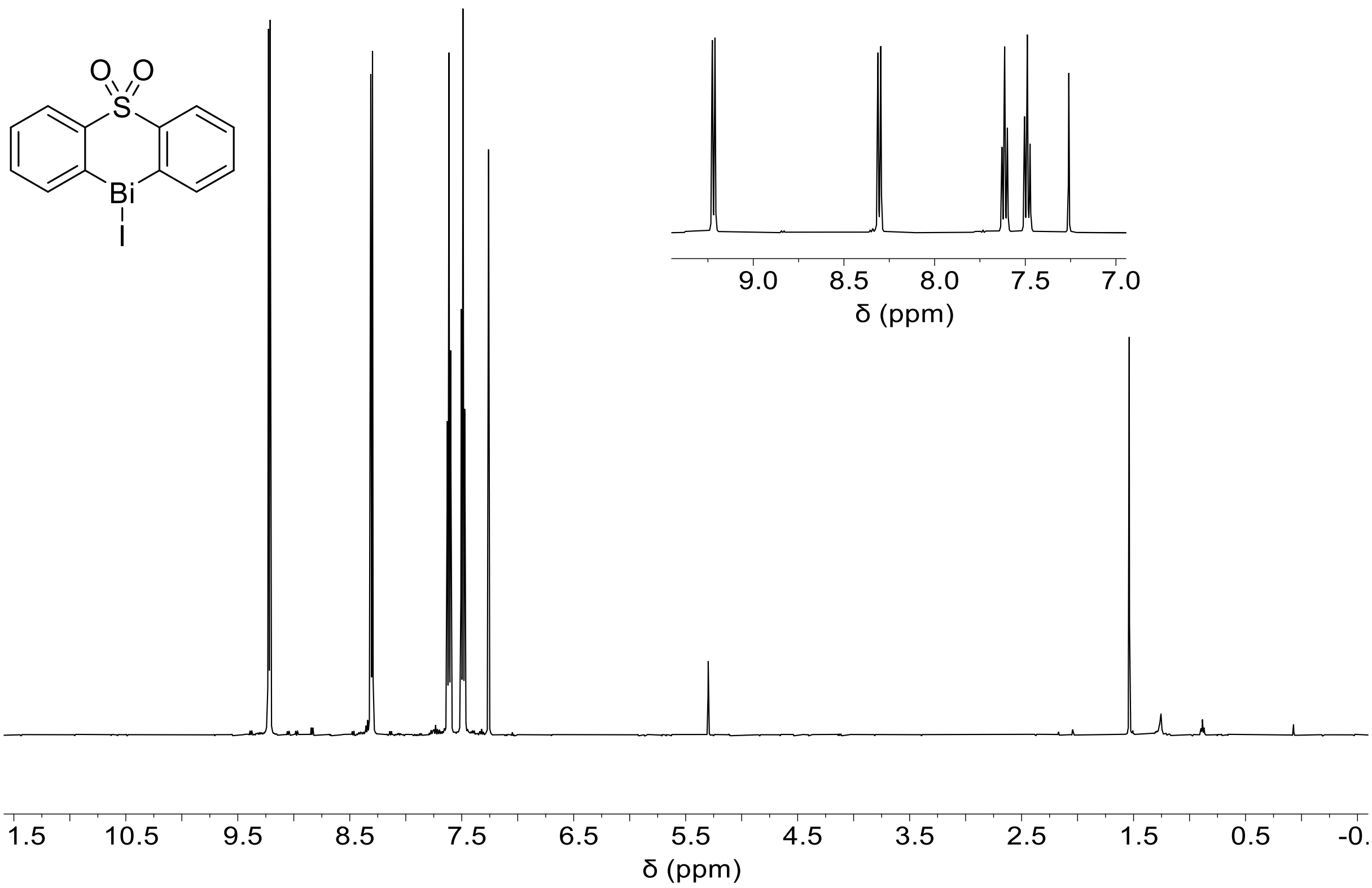

^{1}H NMR (500 MHz) spectrum of **1** in $CDCl_3$.

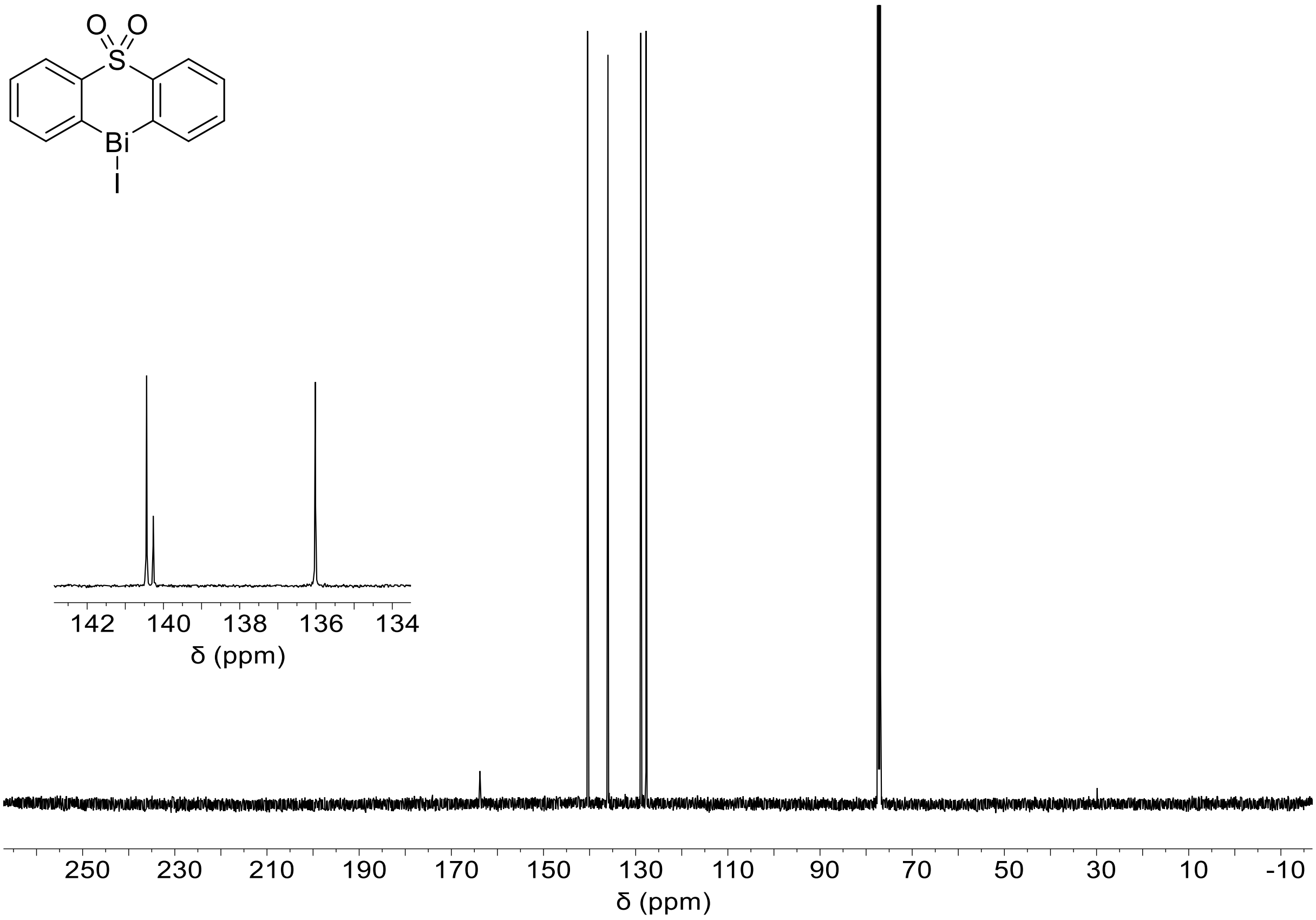

^{13}C NMR (126 MHz) spectrum of **1** in $CDCl_3$.

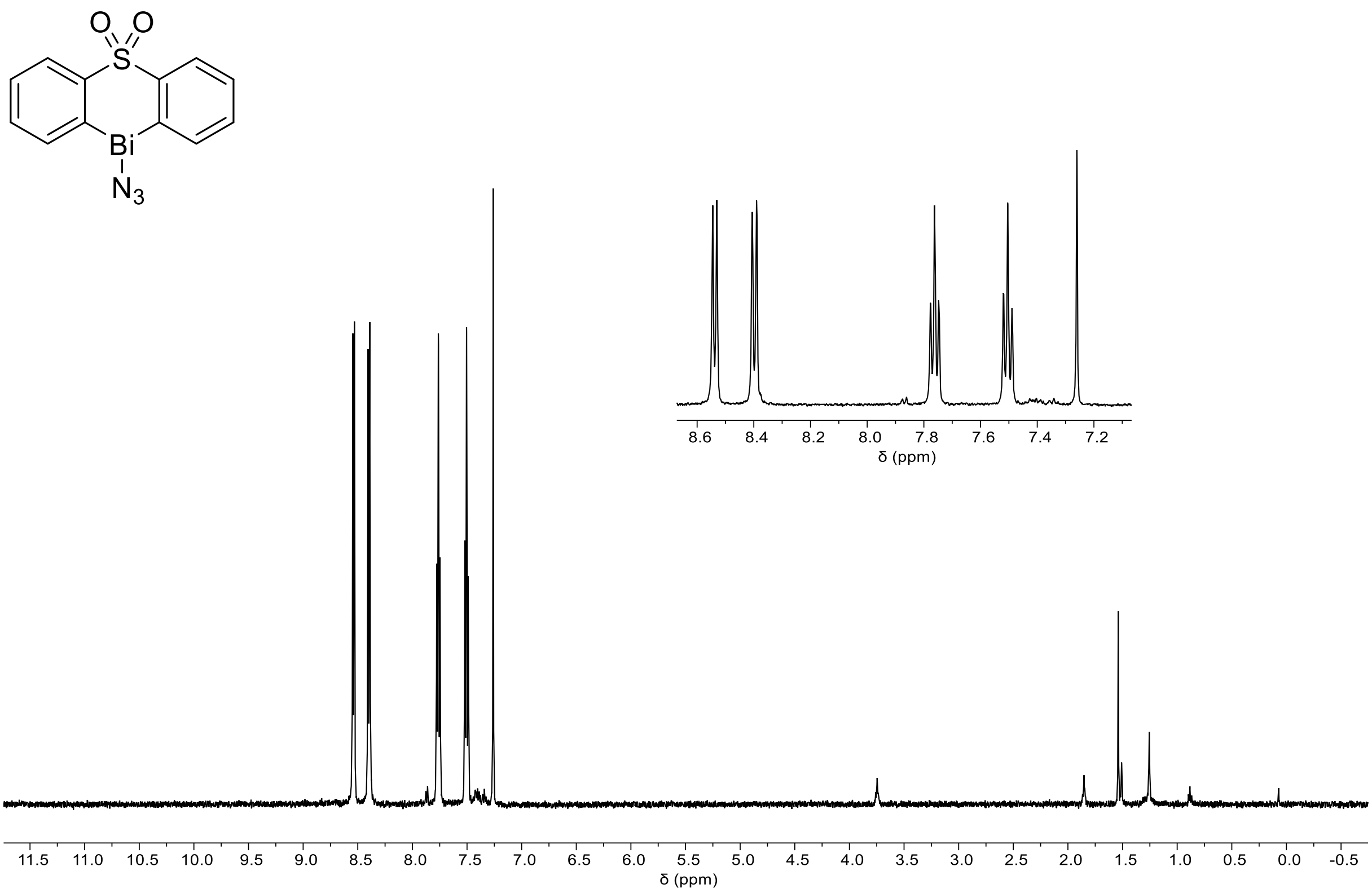

^{1}H NMR (500 MHz) spectrum of **2** in $CDCl_3$.

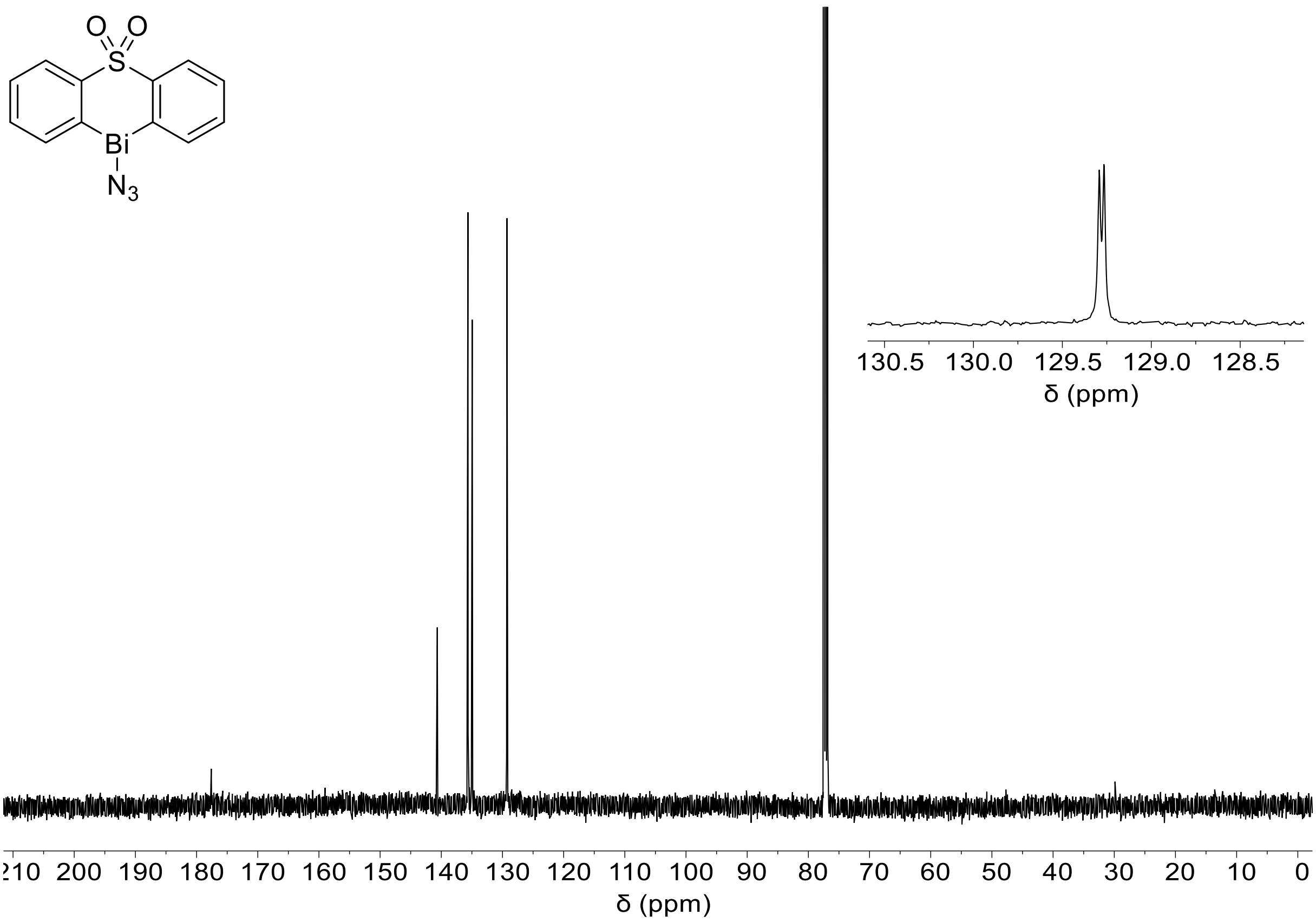

^{13}C{H} NMR (126 MHz) spectrum of **2** in $CDCl_3$.

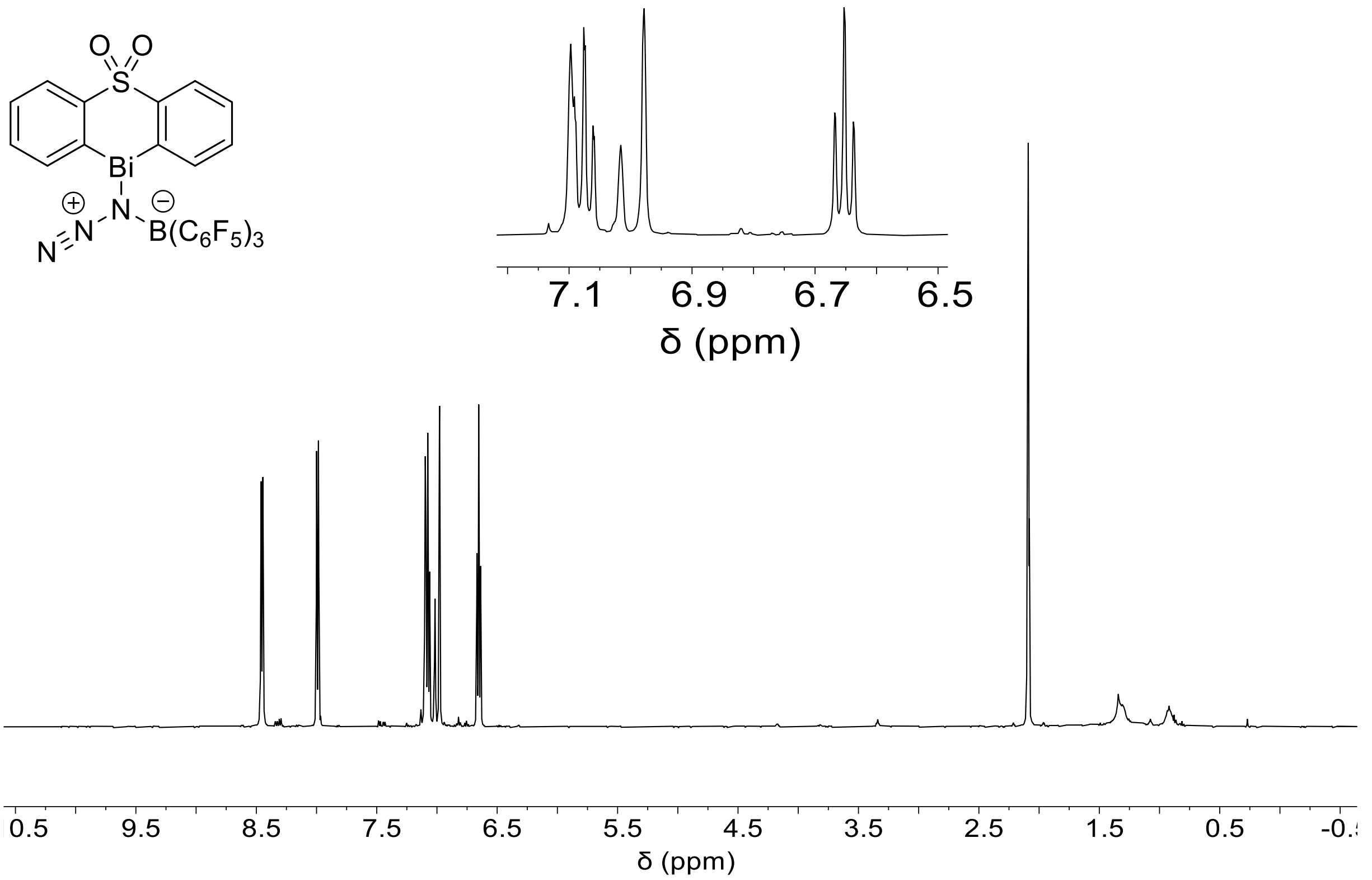

^{1}H NMR (500 MHz) spectrum of **3** in toluene-d_8.

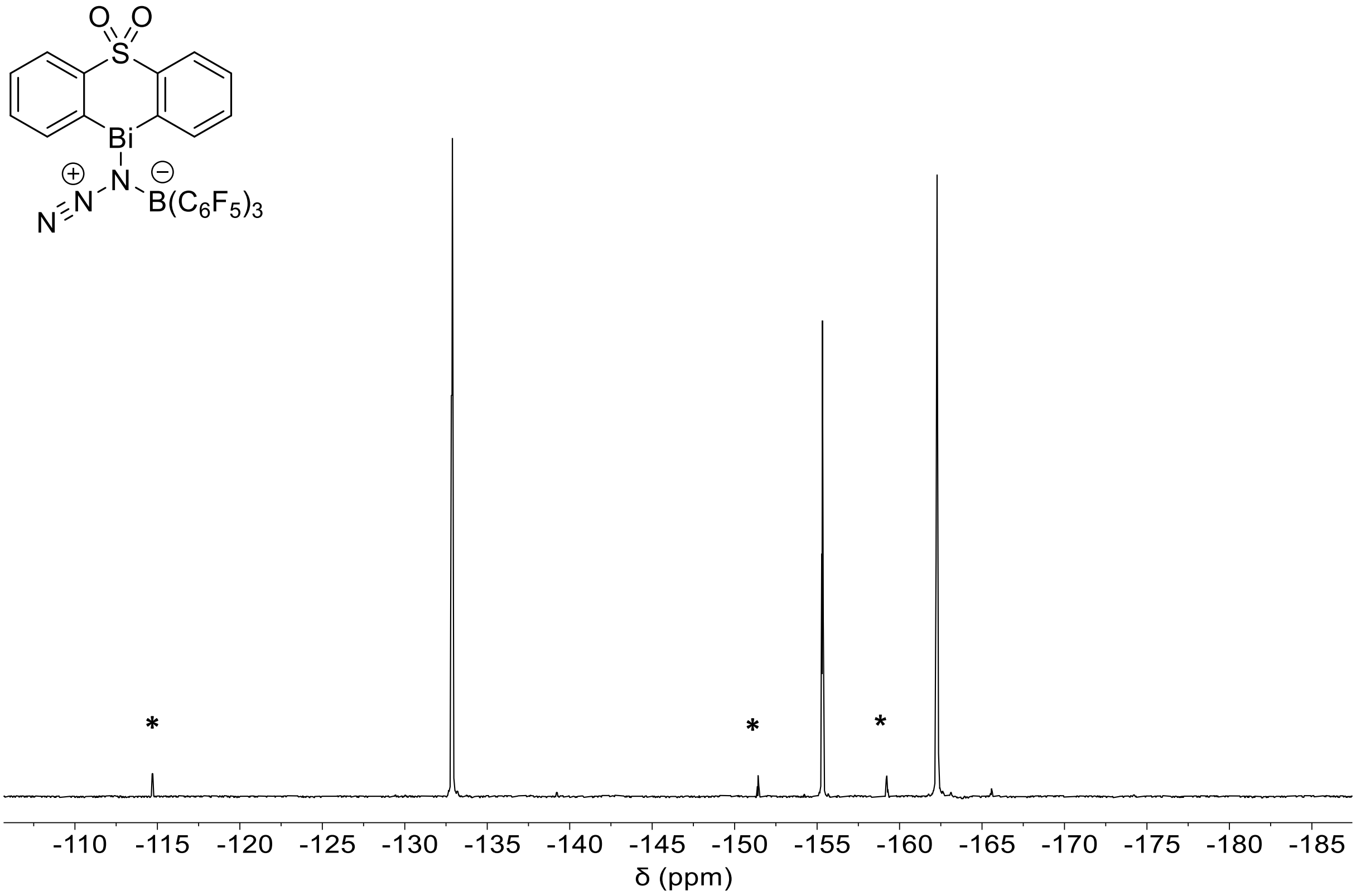

^{19}F{H} NMR (470 MHz) spectrum of **3** in toluene-d$_8$. The peaks marked with (*) are due to the incipient conversion into **5** (~2%) and **4a** (not visible as its concentration is ¼ of **5** due to the reaction stoichiometry).

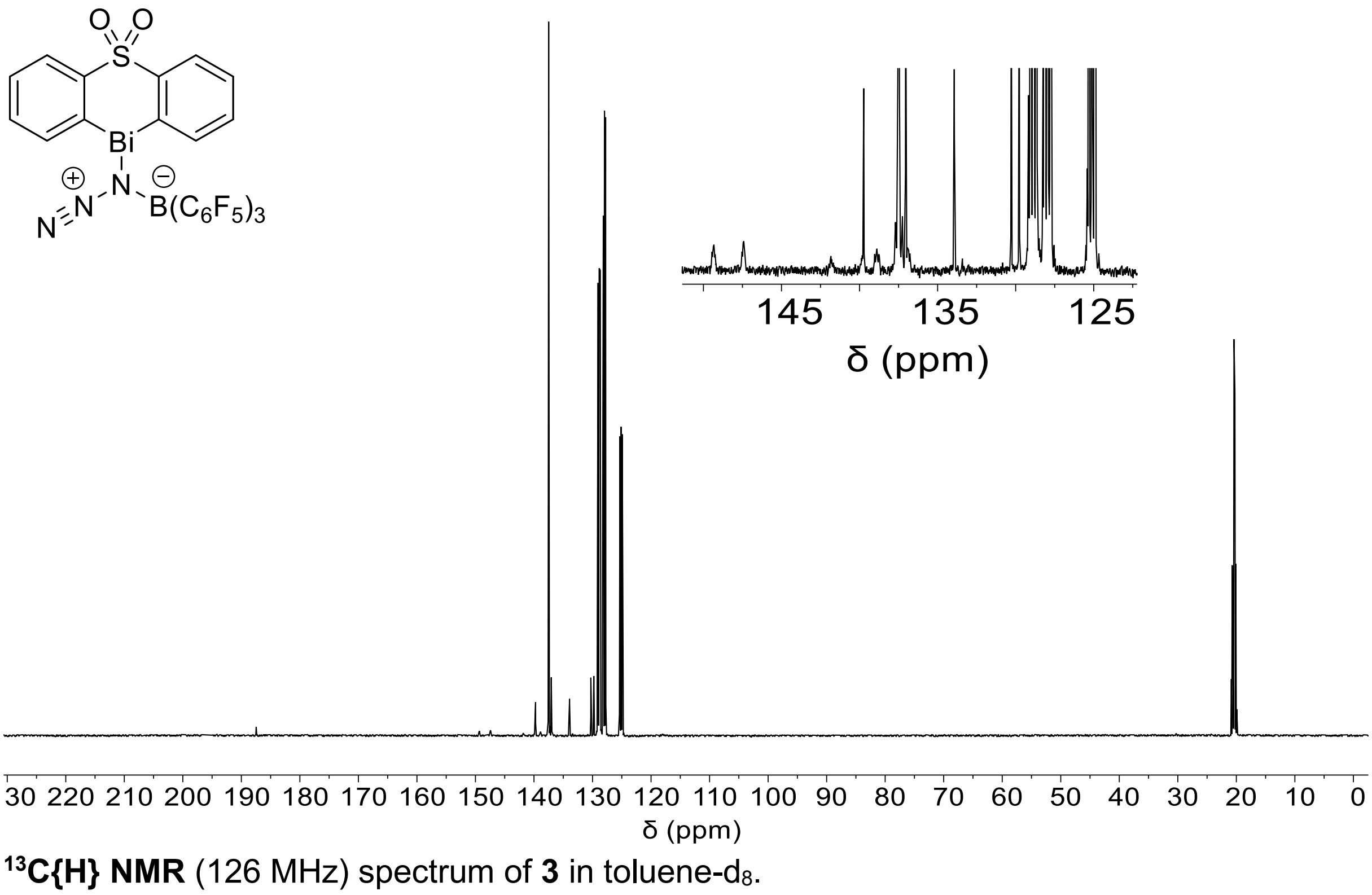

^{13}C{H} NMR (126 MHz) spectrum of **3** in toluene-d$_8$.

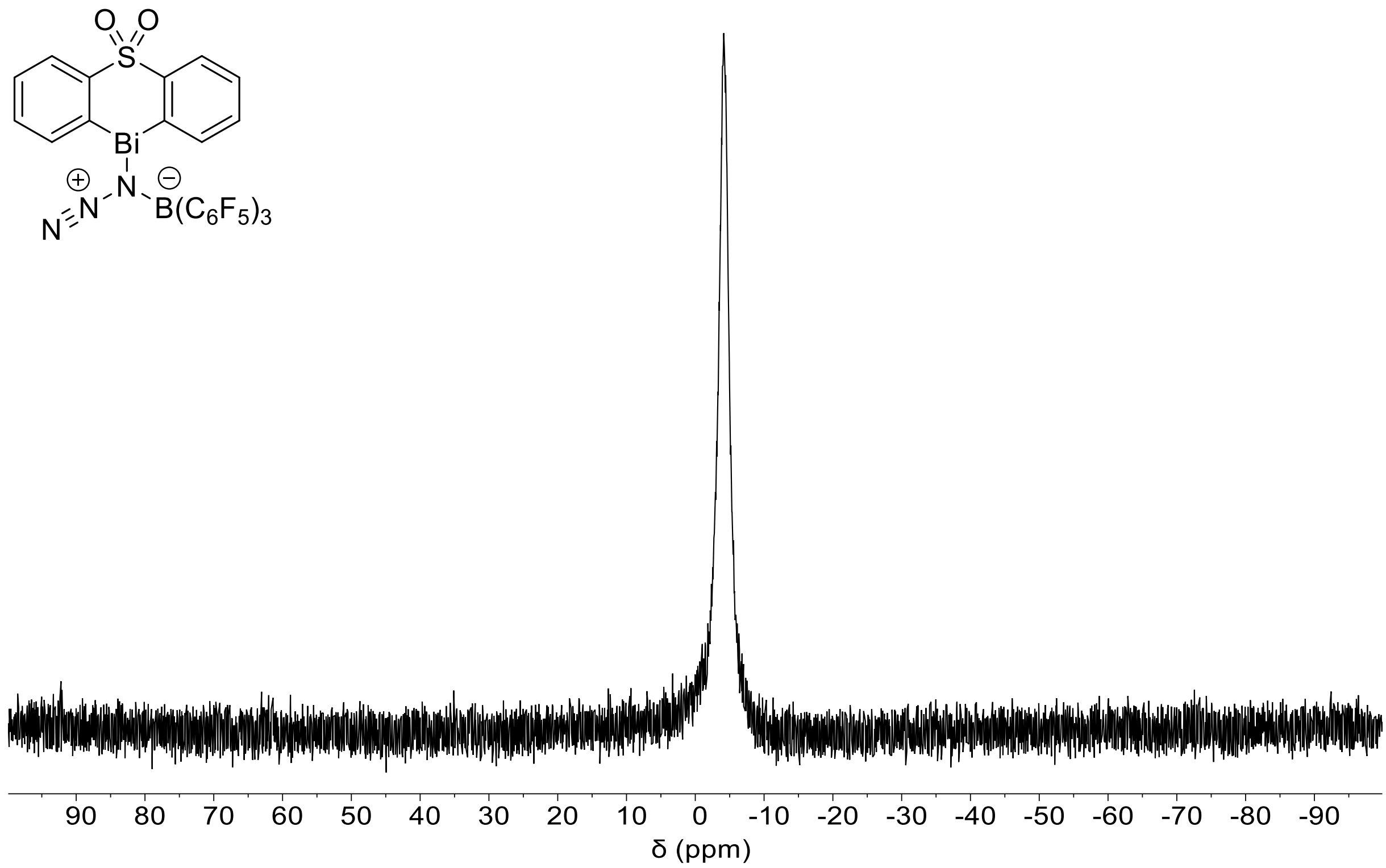

^{11}B NMR (128 MHz) spectrum of **3** in toluene-d_8.

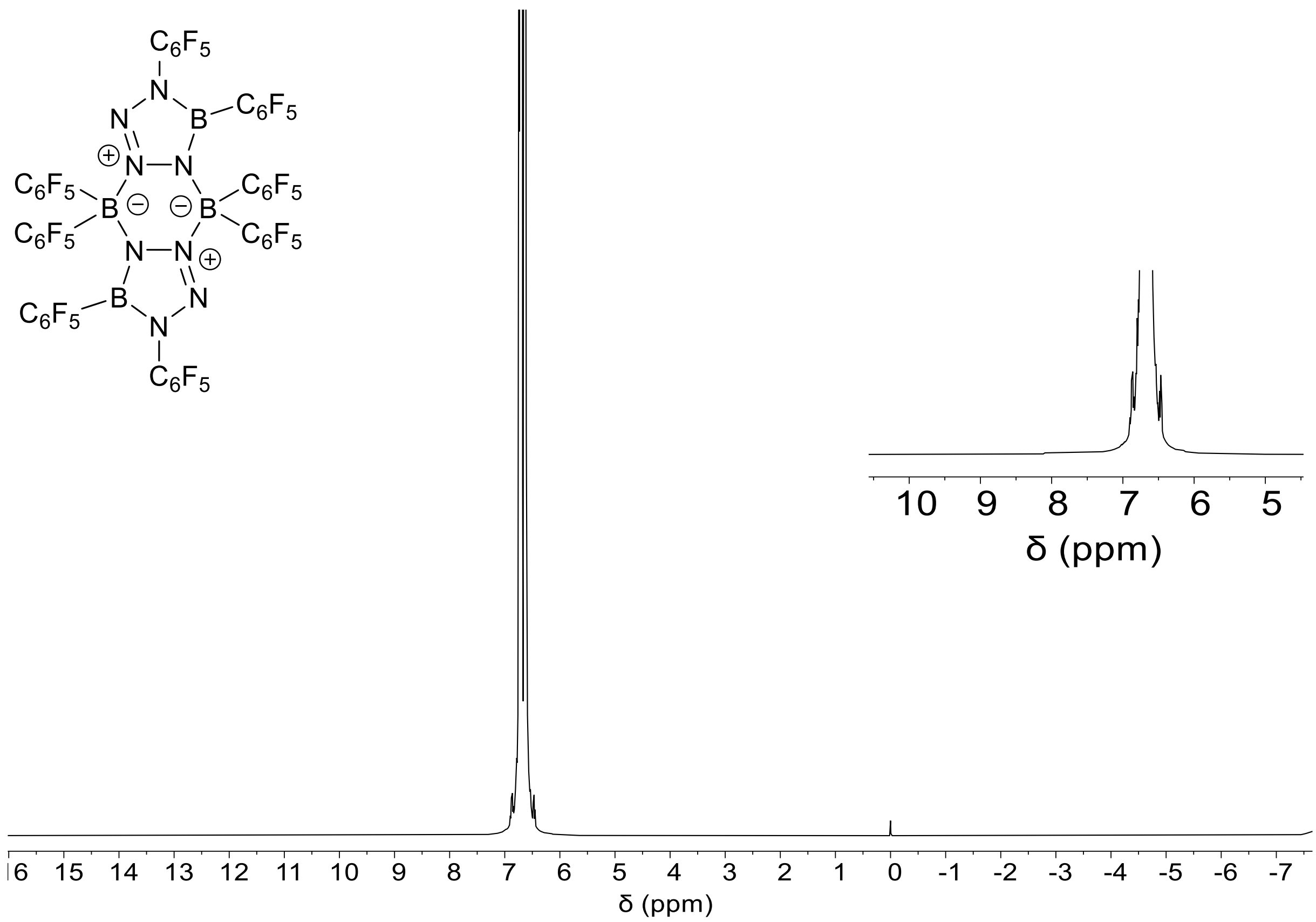

^{1}H NMR (500 MHz) spectrum of **4a** in $C_6H_4F_2$.

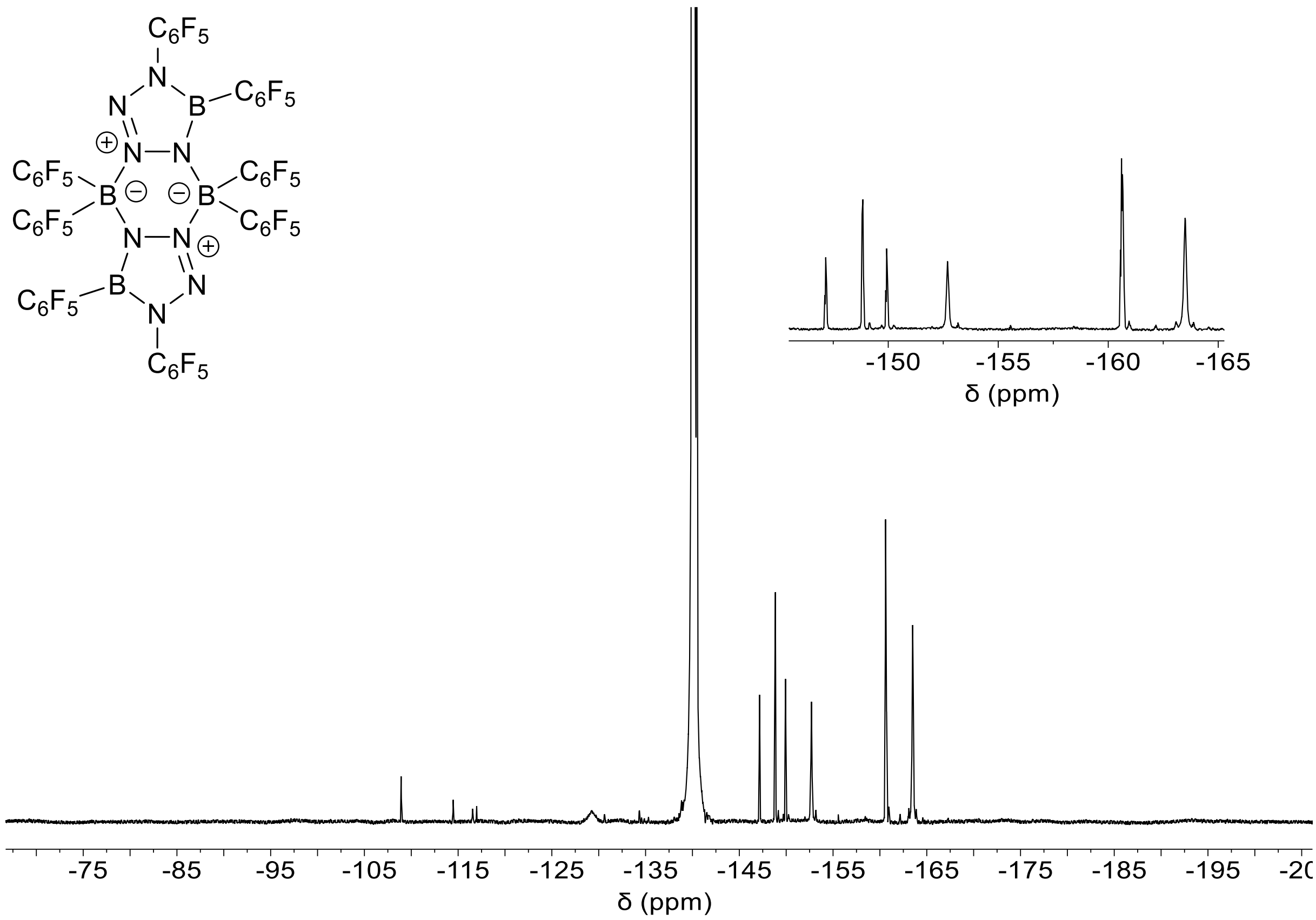

^{19}F{H} NMR (470 MHz) spectrum of **4a** in $C_6H_4F_2$.

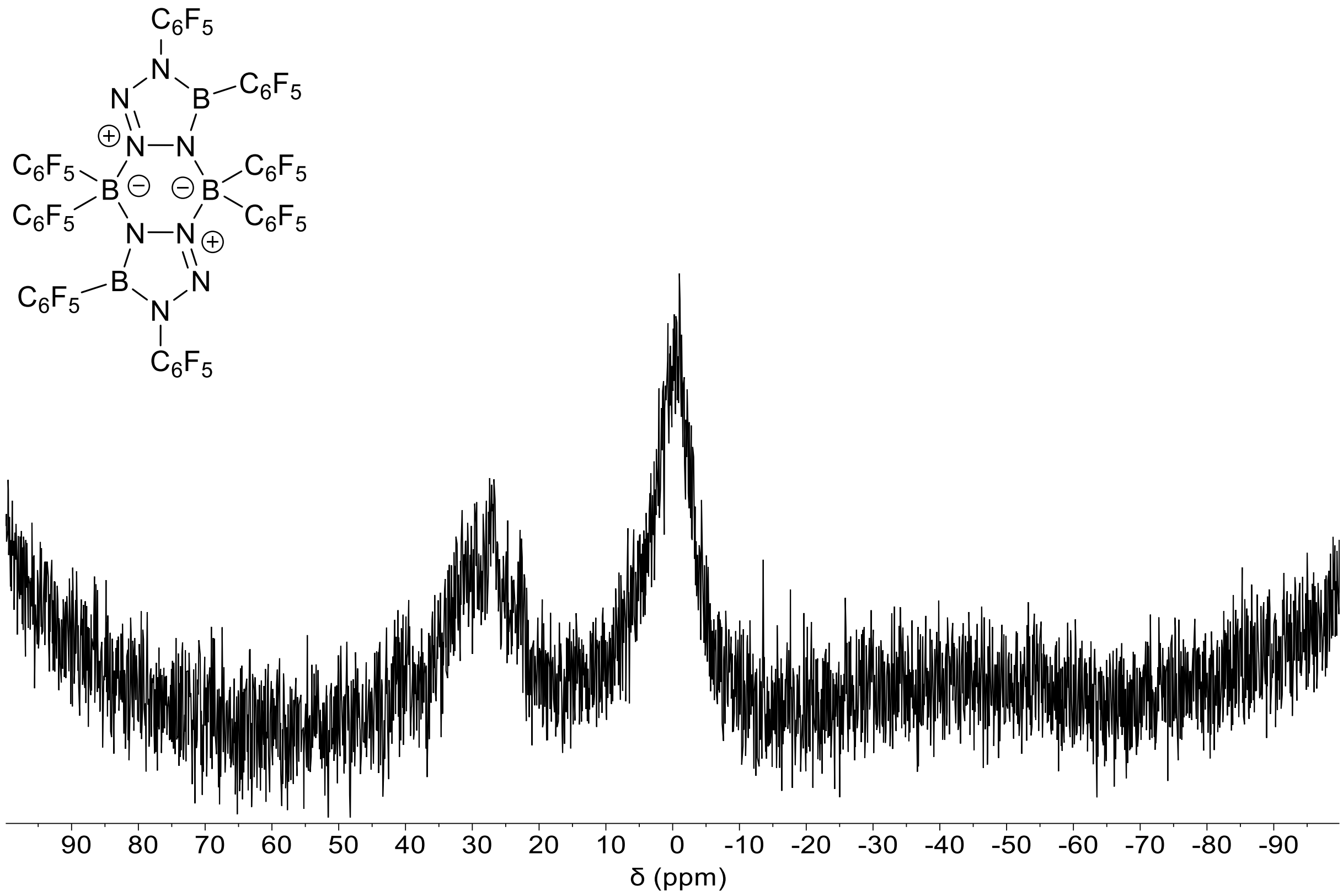

^{11}B NMR (128 MHz) spectrum of **4a** in $C_6H_4F_2$.

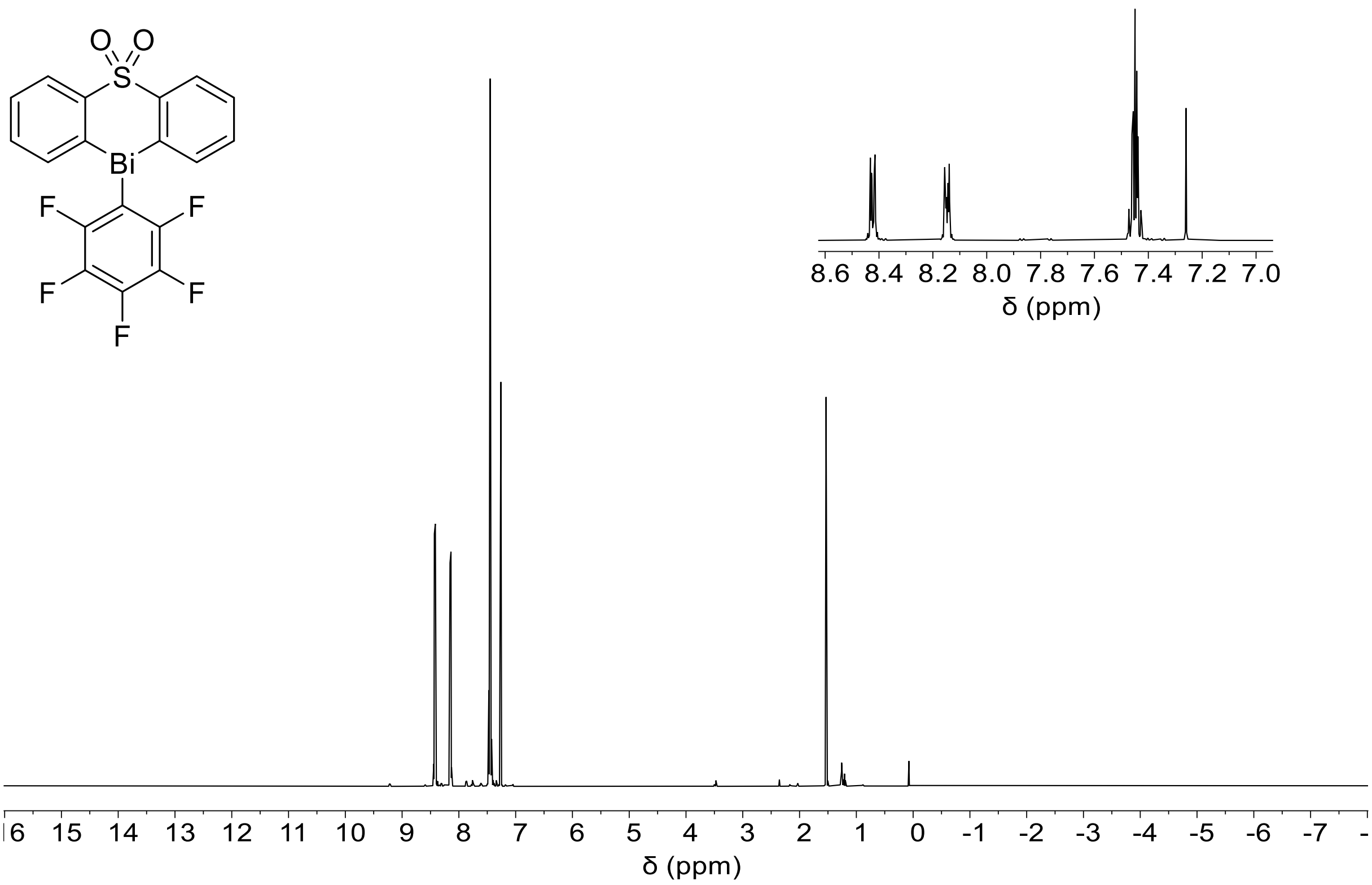

^{1}H NMR (500 MHz) spectrum of **5** in $CDCl_3$.

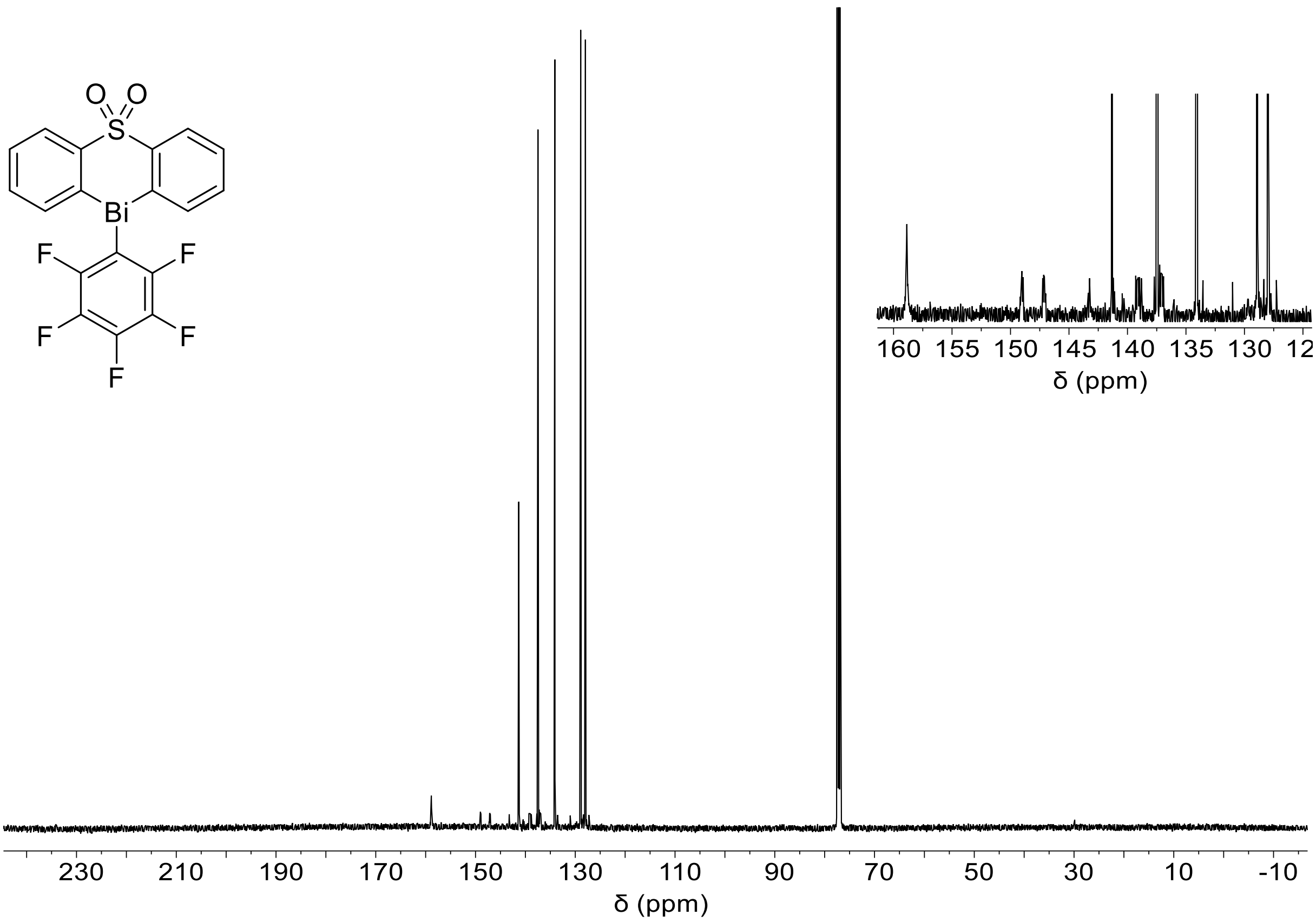

^{13}C{H} NMR (126 MHz) spectrum of **5** in $CDCl_3$.

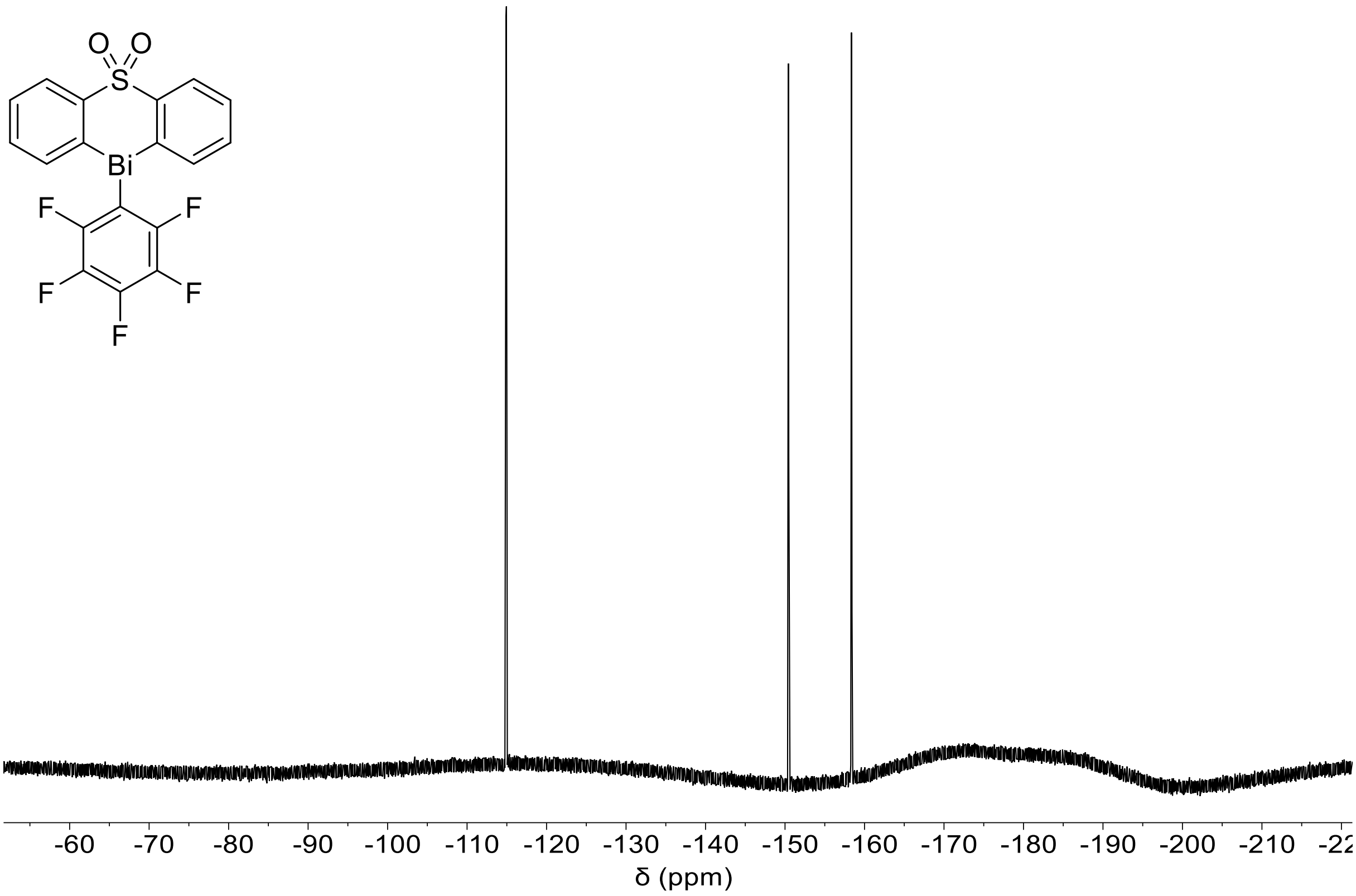

^{19}F{H} NMR (470 MHz) spectrum of **5** in $CDCl_3$.

Single crystal X-ray diffraction analysis

Crystals of **1** were obtained by slow evaporation of a saturated DCM solution at r.T.. Crystals of **2** were obtained from a saturated solution in DCM:*i*-PrOH (2:1 v/v) at -18 °C. Crystals of **3** were obtained via slow diffusion of *n*-hexane into a saturated toluene solution at -30 °C. Crystals of **4a** were obtained by cooling a saturated toluene solution at -30 °C.

A clear greenish, almost colourless plate of **1** (0.28 x 0.16 x 0.10 mm^3) and clear colourless plates of **3** (0.24 x 0.10 x 0.02 mm) were mounted on a Bruker D8 Venture 4-circle Kappa-diffractometer equipped with a low-temperature device (100(2)K, Oxford Cryostream 800er series, Oxford Cryosystems) by using Mo-*K*α-radiation (λ = 0.71073 Å, Helios mirror optics) and a PHOTONIII/C14 CMOS detector system. Intensities were measured by fine-slicing φ- and ω-scans and corrected for background, polarization and Lorentz effects. A semi-empirical absorption correction from equivalent reflections (multi-scan type) was performed by using SADABS.[5]

A clear colourless plank-like specimen of **2** (0.10 x 0.10 x 0.10 mm) and a clear colourless block of **4a** (0.418 x 0.280 x 0.182 mm) were mounted on a STOE IPDS-2T 2-circle diffractometer equipped with a low-temperature device (123(2)K, Oxford Cryostream 700er series, Oxford Cryosystems) by using Mo-*K*α-radiation (λ = 0.71073 Å, ASTIX++-optics) and an image-plate (34 cm diameter) detector system. Intensities were measured by fine-slicing ω-scans and corrected for background, polarization and Lorentz effects. A semi-empirical absorption correction by scaling of reflection intensities with a subsequent spherical absorption correction was performed with LANA.[6]

Structure solution was done using intrinsic phasing methods included in the SHELXT program system[7] and refined by full matrix least-squares/difference Fourier synthesis with ShelXL-2019/3).[8] All non-hydrogen atoms were refined anisotropically; hydrogen atoms were placed in geometrically calculated positions and included using a riding model on the bound carbon atoms and relative isotropic displacement parameters. Olex2 was used for graphical representations of the molecular structures.[9] Specific refinement details are given in the deposited CIF files or below.

CCDC numbers CCDC-2528901 (**1**), CCDC-2528902 (**2**), CCDC-2528903 (**3**), and CCDC-2528904 (**4a**) contain the supplementary crystallographic data for this paper, which can be obtained free of charge from the Cambridge Crystallographic Data Centre via http://www.ccdc.cam.ac.uk/data request/cif.

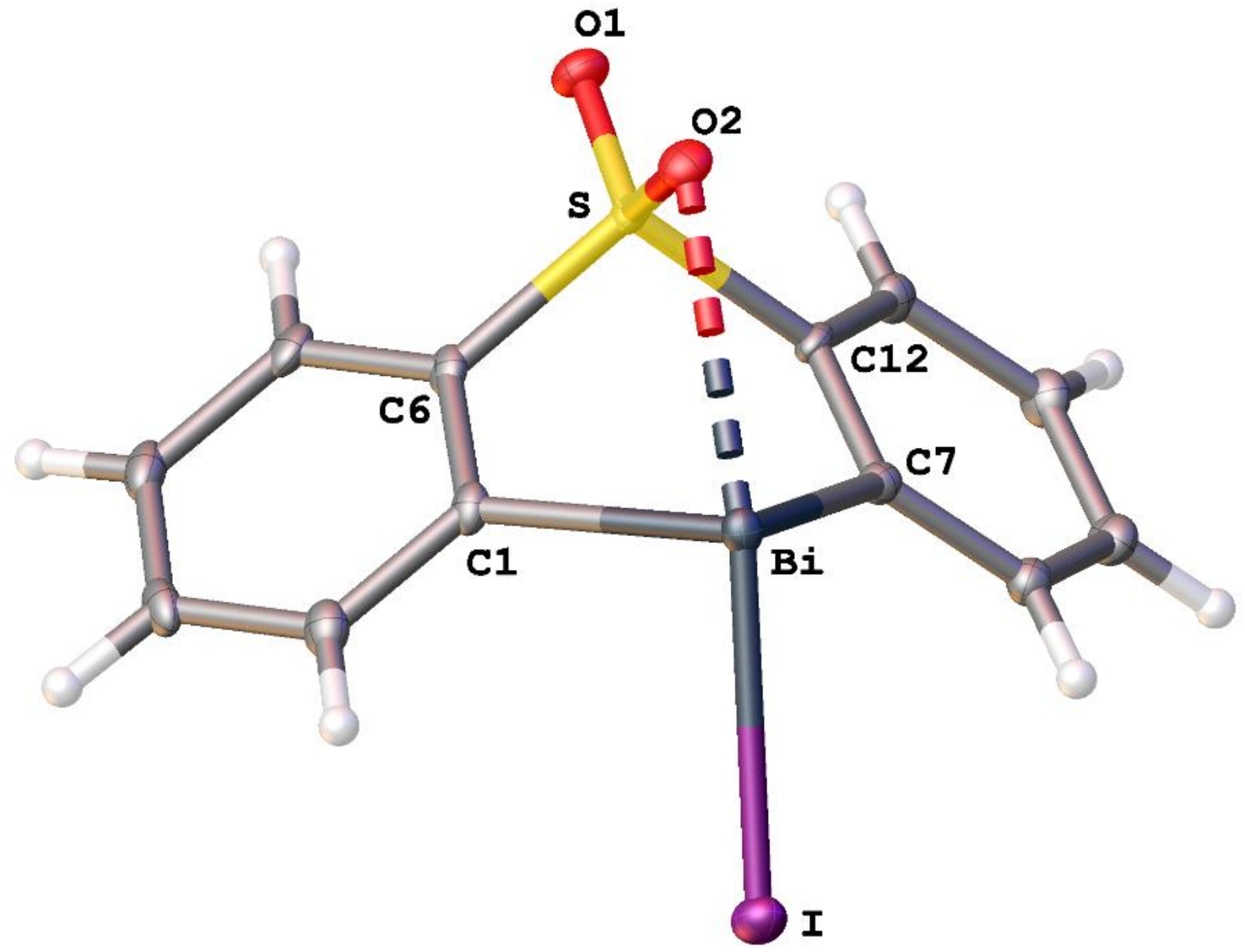

Solid state structure of **1**. Thermal ellipsoids are set to 50% probability level.

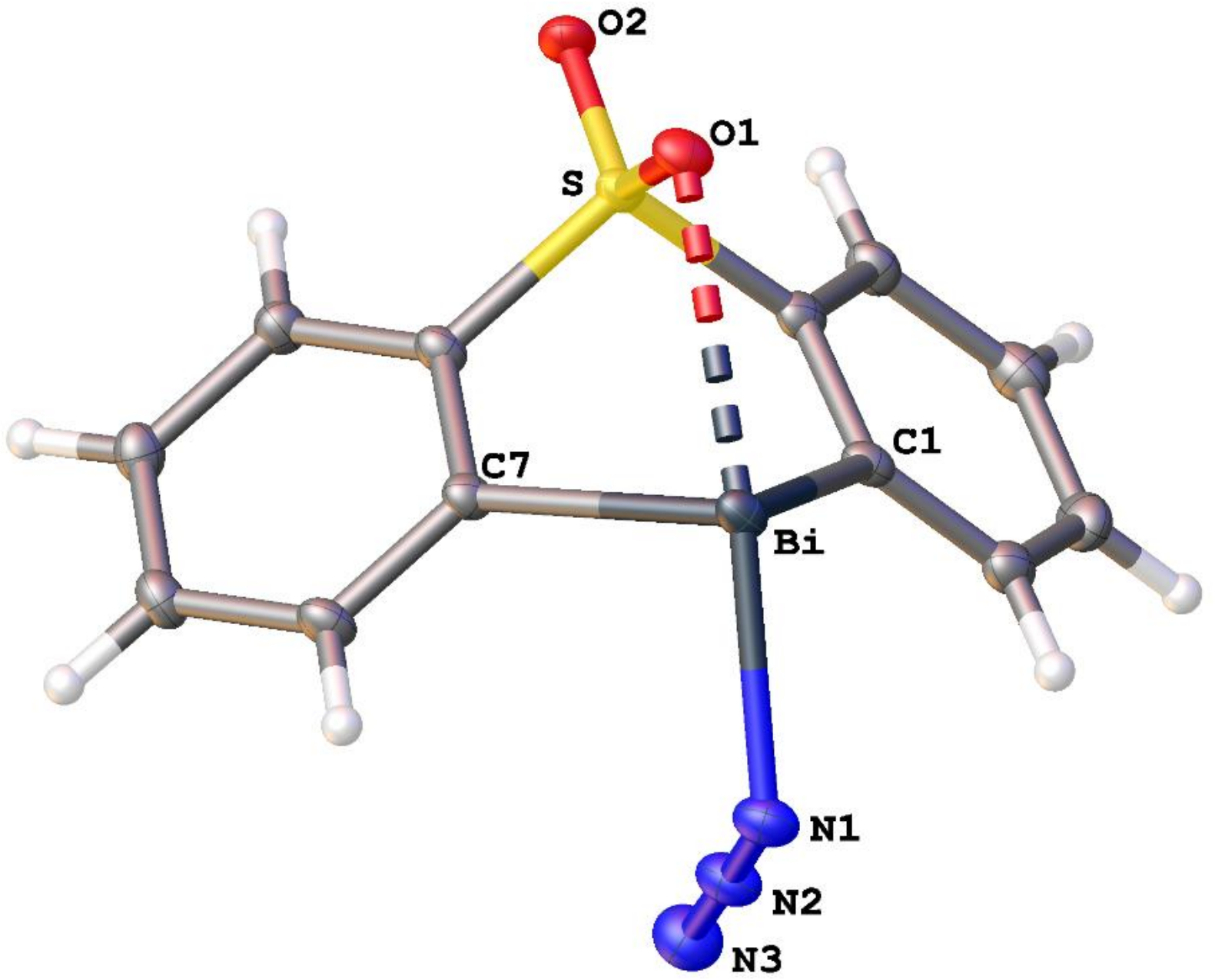

Solid state structure of **2**. Thermal ellipsoids are set to 50% probability level.

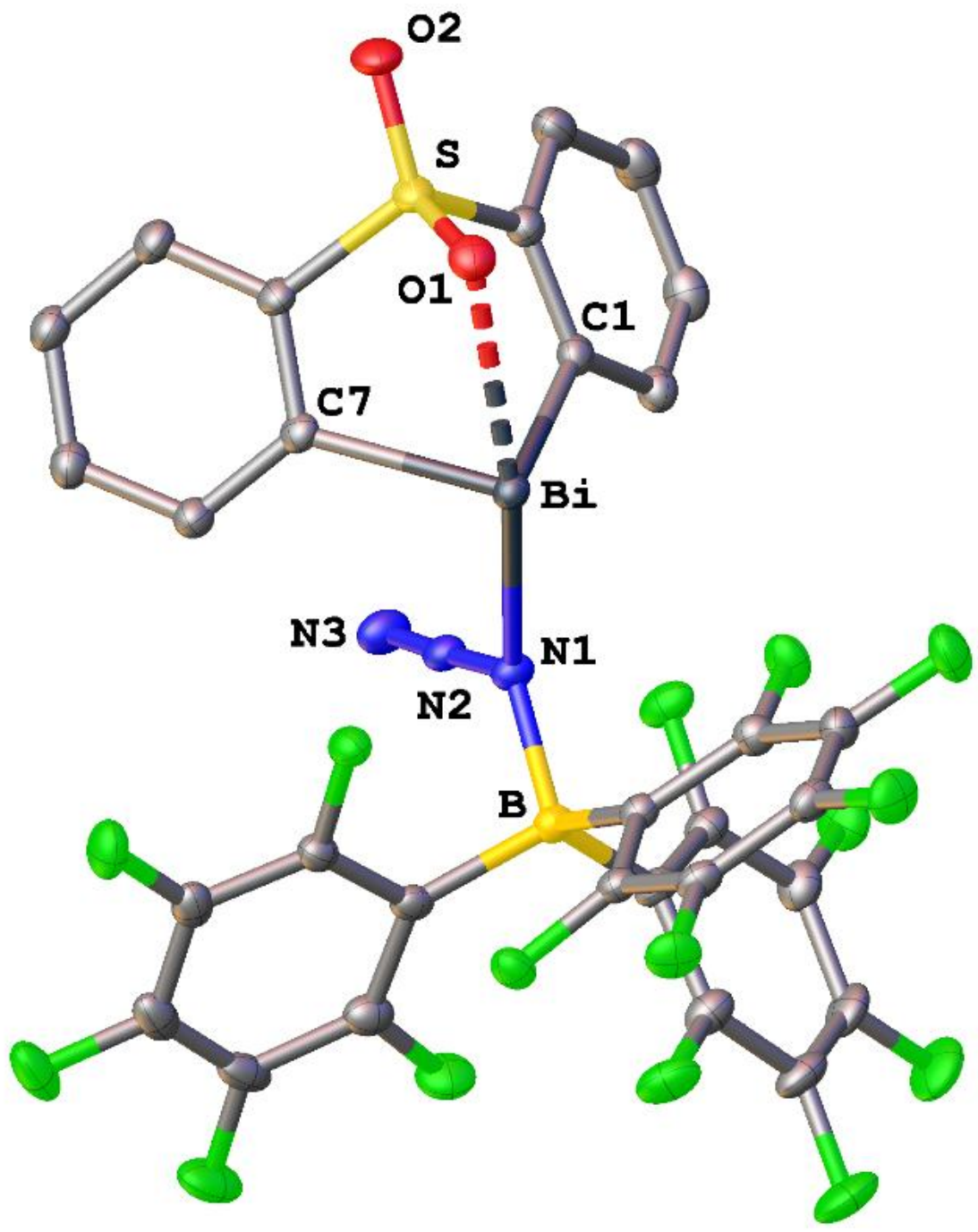

Solid state structure of **3**. Hydrogen atoms are omitted for clarity. Thermal ellipsoids are set to 50% probability level. The contribution of a highly disordered Toluene solvent molecule to the calculated structure factor has been handled by the solvent masking feature as implemented in Olex2.

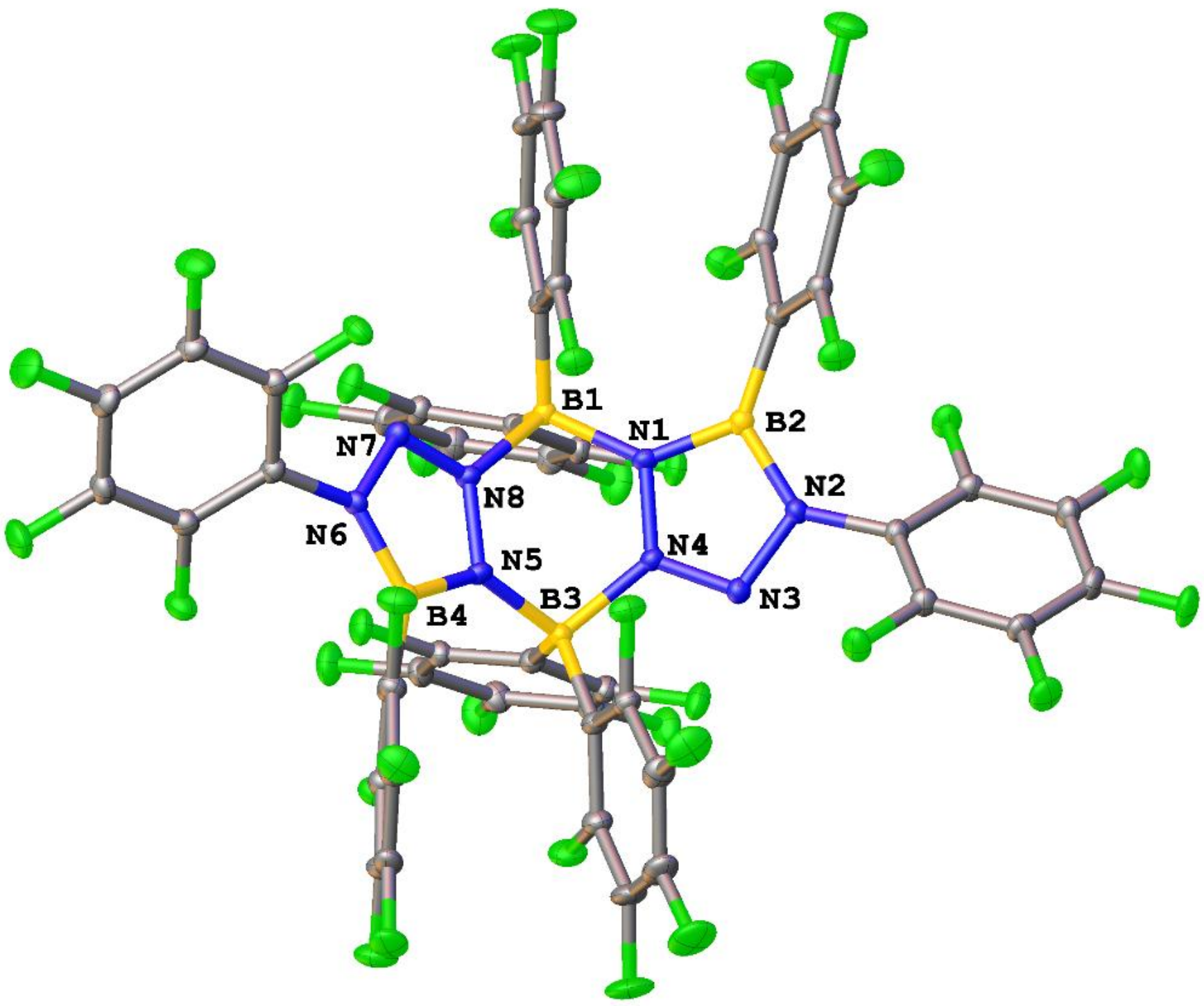

Solid state structure of **4a**. Two co-crystallised independent Toluene solvent molecules have been omitted for clarity. Thermal ellipsoids are set to 50% probability level.

References

1. K. Ruffell, S. P. Argent, K. B. Ling, L. Ball, Bismuth-Mediated α-Arylation of Acidic Diketones with ortho-Substituted Boronic Acids. *Angewandte Chemie International Edition* **2022,** *61* (40), e202210840. https://doi.org/10.1002/anie.202210840.

2. H. Suzuki, T. Murafuji, N. Azuma, Synthesis and reactions of some new heterocyclic bismuth-(III) and -(V) compounds. 5,10-Dihydrodibenzo[b,e]bismine and related systems. *Journal of The Chemical Society-perkin Transactions 1* **1992**, 1593-1600.

3. T. Roy Dennington, J. M. Millam, GaussView 6.1. *Semichem Inc., Shawnee Mission, Kansas* **2016**.

4. S. Grimme, S. Ehrlich, L. Goerigk, Effect of the damping function in dispersion corrected density functional theory. *Journal of Computational Chemistry* **2011,** *32* (7), 1456-1465. https://doi.org/10.1002/jcc.21759.

5. T. A. Teichtmeister, M. M. Hladik, H. Huppertz, High-pressure synthesis and crystal structure determination of Tb2SiB2O8. *Zeitschrift für Naturforschung B* **2023,** *78* (1-2), 25-31. doi:10.1515/znb-2022-0140.

6. J. Koziskova, F. Hahn, J. Richter, J. Kozisek, Comparison of different absorption corrections on the model structure of tetrakis(μ2-acetato)-diaqua-di-copper(II). *Acta Chimica Slovaca* **2016,** *9*. 10.1515/acs-2016-0023.

7. G. Sheldrick, SHELXT - Integrated space-group and crystal-structure determination. *Acta Crystallographica Section A* **2015,** *71* (1), 3-8. doi:10.1107/S2053273314026370.

8. G. Sheldrick, Crystal structure refinement with SHELXL. *Acta crystallographica. Section C, Structural chemistry* **2015,** *71* (Pt 1), 3-8. 10.1107/s2053229614024218.

9. O. V. Dolomanov, L. J. Bourhis, R. J. Gildea, J. A. K. Howard, H. Puschmann, OLEX2: a complete structure solution, refinement and analysis program. *Journal of Applied Crystallography* **2009,** *42* (2), 339-341. doi:10.1107/S0021889808042726.
